# Exploration of the anti-inflammatory potential of *Polygonum bistorta* L.: protection against LPS-induced acute lung injury in rats via NF-ĸβ pathway inhibition

**DOI:** 10.3389/fphar.2024.1500085

**Published:** 2025-02-05

**Authors:** Sajida Parveen, Kashif ur Rehman Khan, Shahid Muhammad Iqbal, Hanan Y. Aati, Areej M. Al-taweel, Liaqat Hussain, Musaddique Hussain

**Affiliations:** ^1^ Department of Pharmacology, Faculty of Pharmacy, The Islamia University of Bahawalpur, Bahawalpur, Pakistan; ^2^ Department of Pharmaceutical Chemistry, Faculty of Pharmacy, The Islamia University of Bahawalpur, Bahawalpur, Pakistan; ^3^ Michael Sars Center, University of Bergen, Bergen, Norway; ^4^ Department of Pharmacognosy, College of Pharmacy, King Saud University, Riyadh, Saudi Arabia; ^5^ Department of Pharmacology, Faculty of Pharmaceutical Sciences, Government College University Faisalabad, Faisalabad, Pakistan; ^6^ Department of Medicine, University of Alabama at Birmingham, Birmingham, AL, United States

**Keywords:** polygonum bistorta, polygonaceae, NF-ĸβ, gene expression, medicinal plants, molecular docking

## Abstract

Traditional medicine uses the roots and rhizomes of *Polygonum bistorta* L. (Polygonaceae) to treat cough, bronchitis, and other respiratory infections. Our goal was to gain insights into the lung protective effects of the roots of *P. bistorta* L. against lipopolysaccharide-induced acute lung injury in rats, along with the possible mechanism(s). The outcomes revealed deliberate quantities of the total phenolic and flavonoid contents of 156.2 ± 5.13 GAE/g and 179.45 ± 2.08 mg QE/g, respectively. Crude extract possesses a maximum inhibitory potential of 81.77% ± 0.62% for acetylcholinesterase against eserine. Acute oral toxicity study revealed LD_50_ beyond 7 g/kg. Plant extract markedly restored LPS-induced hypoxemia, pulmonary edema, histopathological alterations, and leukocyte infiltration in the lung. ELISA testing on BALF found that the plant extract efficiently reinstated superoxide dismutase, total anti-oxidant capacity, malondialdehyde, and total oxidative stress. qRT-PCR indicated a decline in the endotoxin-induced overproduction of pro-inflammatory markers, oxidative stress, transcription factor, and downregulated antioxidant potential in extract-treated groups. Furthermore, 24 metabolites were identified and quantified via GC-MS. A molecular docking procedure was implemented on the bioactive metabolites that were identified to evaluate their potential for inhibiting AChE. In conclusion, *P. bistorta* roots mitigate inflammation and oxidative stress by improving redox signaling and NF-ĸβ (p65) pathways and can thus play a role in strategies for overcoming therapeutic challenges.

## Graphical Abstract

**Figure:**
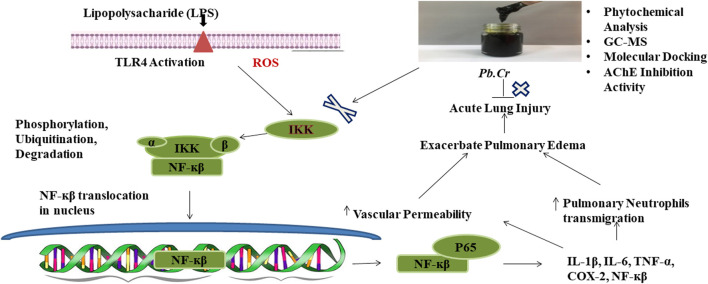

## 1 Introduction

Despite much advanced research, even with a decreasing death rate, acute lung injury (ALI) continues to be a major cause of morbidity and death for severely ill patients (Mowery et al., 2020). ALI or its extreme description, severe acute respiratory distress syndrome (ARDS), is a devastating lung disease that has an elevated rate of complications throughout the world (Mowery et al., 2020; Yu et al., 2021; Butt et al., 2016). ALI/ARDS is characterized by lung edema and hypercapnia caused by elevated alveolar-capillary membrane leakage (EACML) and the consequent reduction of arterial oxygenation (Ragaller and Richter, 2010; Mokra and Kosutova, 2015; An et al., 2019). The overall fatality rate of ALI is higher in the United States (Butt et al., 2016), at more than 55 per 100,000 cases annually, than in Australia, Europe, and other advanced nations, which have more than 27 per 100,000 cases (Goyal et al., 2012). ALI and ARDS account for almost 10% of all intensive care unit (ICU) cases and four percent of all hospital admissions. Two major causes of ALI are direct and indirect injuries. Microbial infections, lung contusions, pulmonary embolism, and near drowning are direct causes of ALI/ARDS, whereas sepsis, pancreatitis, trauma, and drug overdoses are categorized as indirect causes (Hussain et al., 2021). Understanding the pathophysiology of ALI has led to the identification of various biochemical indicators linked to non-satisfactory clinical outcomes. Events that either lead or predispose to ALI/ARDS often involve three interrelated pathophysiologic conditions: (i) increased microvascular permeability, (ii) alveolar instability reducing lung compliance, and (iii) overactive and disrupted lung inflammatory responses like interleukins (IL-1, IL-6), chemokines, tumor necrosis factor (TNF-α), oxidants (superoxide ions, hydrogen peroxide, nitric oxide), and proteases, which escalate inflammation by destroying alveolar architecture (Matuschak and Lechner, 2010).

Randomized clinical trials have found no particular drugs to be beneficial in treating ALI/ARDS (Calfee and Matthay, 2007). Although there is a paucity of data on outcomes, supportive care comprising mechanical respiration, positive end-expiratory pressure (PEEP), smaller tidal volumes, a conservative fluid approach, and lower inhalation pressures have slightly improved survival rates (Halter et al., 2007). Several intriguing novel treatments are being studied in current or planned clinical trials, such as nitric oxide, corticosteroids, heparin, surfactants, ibuprofen, azithromycin, activated protein C, β_2_-agonists, lysofylline, phosphodiesterase inhibitors, neuromuscular blockers, aspirin, anti-TNF biologics, and antioxidants ((Mullen et al., 1993; Teke et al., 2008; Tanaka et al., 2008; Husari et al., 2014; Zhang et al., 2014; Kumari et al., 2015; Mokra and Kosutova, 2015; Kosutova et al., 2016; Meng, 2017; Dixon et al., 2021; Muhammad et al., 2023;; Rajasekar et al., 2024). Studies suggest that albumin and furosemide administration may only provide symptomatic relief in hypoproteinemic individuals suffering lung injury (Calfee and Matthay, 2007). However, no treatments have been demonstrated that enhance patient outcomes in therapeutic cost, quality of life, extent of morbidity, and mortality (Villar et al., 2020). There is thus great demand to develop new and efficient medications for the management of ALI/ARDS, considering its prevalence and poor prognosis (Alamgeer et al., 2018).

The intra-tracheal instillation of endotoxin (lipopolysaccharide) is extensively used to trigger ALI in test animals (Rojas et al., 2005). The pulmonary parenchymal cells are impaired by the discharge of reactive species, activated macrophages in the lung, and transmigrated neutrophils in the alveolar and interstitial sections. The endpoints are microvascular injury and diffuse alveolar damage (DAD) through hemorrhage within the lungs, swelling, neutrophil infiltration, deposition of fibrin, and complementary system activation (Triggianese et al., 2023; Shi et al., 2023). Pro-inflammatory markers were also released, including interleukins-1β, 6, and TNF-α, which enhance the level of transcription factor NF-ĸβ. ALI/ARDS has been induced using LPS, a fundamental element of Gram-negative bacteria. Animal models caused by LPS offer valuable insights into the mechanisms behind many diseases and can be used to identify new biomarkers and therapeutic targets (Chen et al., 2010).

Traditional medicinal plants contribute to combating a wide range of respiratory illnesses (Cock and Van Vuuren, 2020; Teka and Maryo, 2023). We can evaluate all traditional remedies that contain bioactive metabolites to create modern pharmaceuticals. Bioactive compounds such as polyphenols and flavonoids have anti-inflammatory, anti-oxidant, antibacterial, and anti-proliferative activities. Over 50% of all current drugs originate from natural sources, so researchers concentrate on drug discovery from botanical preparations (Singh et al., 2020). Considering the results of different biomedical and pharmacological studies, a variety of metabolites like flavonoids (Peng et al., 2019; Shokry et al., 2022), phenolics (Xing et al., 2019), alkaloids (Renushe et al., 2022), saponins (Lin et al., 2016), and terpenoids (Wang et al., 2022) have been suggested for the treatment of ALI (Georgiev et al., 2022). These botanical drugs have diverse anti-inflammatory and anti-oxidative potential and are a step toward novel drug discovery in pulmonology. Numerous studies on different plants have been shown to significantly reduce ALI manifestations, such as a decline in neutrophils infiltration and toll-like receptor pathways (TLR_4_, NF-ĸβ, COX-2) and, consequently, TNF-α, Interleukins (IL-1, -6, -8, -10) and chemokines by reducing oxidative stress in experimental animals (Aboushanab et al., 2021; Liou et al., 2017; Baradaran Rahimi et al., 2019; Shokry et al., 2022; Feng et al., 2019; Tian et al., 2019a; Erdenechimeg et al., 2017; Feng et al., 2024; Tian et al., 2019b; Xing et al., 2019).

The botanical description of *Polygonum bistorta* L. is of a perennial herbaceous plant belonging to the family Polygonaceae. Its other scientific names are *Bistorta officinalis* Delarbre, *Polygonon bistortum* (L.), and *Persicaria bistorta* (L.). Its non-scientific names include adderwort, anjabar, asar-rai, bijband, bandak, ban-natia, dragon wort, hozar, meadow bistort, quanshen, snakeweed, stor-ormrot, wiesen-knoterich, serpentaire, schlangen-wiesenknoterich, and rhizomata bistortae. The plant is native to Pakistan, Turkey, China, and Afghanistan, as well as North America and Europe (Demiray et al., 2009; Klimczak et al., 2017). The botanical drug contains chlorogenic acid, gallic acid, catechin, procyanidin, caffeic acid, quercetin, luteolin, apigenin, and kaempferol. Among the identified physiologically active substances in the extract are various triterpenoids, phenolic acids, flavan-3-ols, flavonols, tannins, and fatty acids (Mehar and Tabarak, 2013; Klimczak et al., 2017). Their antimicrobial, antioxidant, hemostatic, immunostimulatory, anti-inflammatory, hepatoprotective, gastroprotective, and anticancer properties are all attributed to these metabolites (Liu et al., 2006). The roots of *P. bistorta* contain polysaccharides, polyphenols (anthraquinones, stilbenes, naphthalene), flavonoids (flavonols, 2, 5′,6′trihydroxy-4,2′-dimethoxy flavones, and 2, 3′,4′,4,6-pentahydroxy flavones, kaempferol, myricetin, arborinone, adeaninone, baicalin, arborinol, luteolin, rhamnetin), triterpenoids (24(E)-ethylidenecycloartanone and 24(E)-ethylidenecycloartan-3-ol), steroids, tannins (1-galloyl-β-d-glucose, castalagin, proanthocyanidin), catechins (epicatechins, protocatechuic, and gallocatechin), and glycosides (Manoharan et al., 2005; Orbán-Gyapai et al., 2015; Pillai et al., 2019; Pirvu et al., 2017). Another novel metabolite, 3-methyl-gallic acid 4-*O*-β-d-(6′-*O*-3″-methyl-galloyl)-glucopyranoside (isoquercetin), was elucidated from bistorts by Liu et al. (2006). Research has reported the presence of quercimeritrin, avicularin, rutin, hyperine, mequelianin, isoquercetin, spirozide, cyanidine, anthocyanidine, delphinidine, myristic, palmitic acid, furfural, linoleic acid, and oleic acid in bistorts (Sun et al., 2007; Intisar et al., 2013; Dhiman et al., 2023).

In folklore, phytomedicine (Javid et al., 2024) is enriched with a high content of polysaccharides and polyphenolics (Klimczak et al., 2017; Pirvu et al., 2017). Aerial parts of this plant are used in dock and savory puddings (Klimczak et al., 2017). According to British, European, Unani, and Chinese pharmacopoeias, the roots and rhizomes are consumed as fluid extract and dried in whole or fragmented forms. Traditionally, this medicinal plant is used to treat cough, bronchitis, severe respiratory infections (Javid et al., 2024), for other inflammatory diseases like acute gastritis, hemorrhoidal bleeding, and nasal bleeding (Munir et al., 2014; Karuppiah Pillai, 2021), as an expectorant, emetic, febrifugal (Mehar and Tabarak, 2013), and as an antidote for measles, smallpox, and snake bite. The reported pharmacological activities of this plant are anti-inflammatory (Pawłowska et al., 2020), antibacterial, (Intisar et al., 2012), antioxidant (Demiray et al., 2009), anti-microbial (Munir et al., 2014; Cecotti et al., 2012) anti-proliferative (Mittal et al., 2013), gastroprotective, anticancer (Khushtar et al., 2018), antipyretic (Kumar et al., 2012), and hepatoprotective (Mittal et al., 2013). The exploration of bistort extract has facilitated the identification of novel bioactive compounds, the assessment of their healing potential, and the assurance of their safety in the management of socially significant diseases. The object of this study was to examine the lung protective effects of an aqueous methanolic extract of the roots of *P. bistorta* L. against LPS-induced ALI in a murine model, as well as potential mechanism(s) of action, in context with the potential benefits of this traditionally used plant.

## 2 Materials and methods

### 2.1 Plant collection and extraction

This experiment was conducted at the Department of Pharmacology of the Islamia University Bahawalpur (IUB). Two kilograms of *P. bistorta* root were procured from a botanical specialist, Rahim Yar Khan of Punjab, Pakistan and identified by a taxonomist, Dr. Abdul Hameed; a dried root sample (Specimen No. Pb. Ro. 06–12-50) was placed in the botanical collection of the Pharmacology Research Lab, IUB Pakistan. After that, all debris, including hair, pollen, dust, and other possible contaminants were removed, the root was chopped into 4–5 cm pieces, and then powdered into a coarse substance. It was soaked in an aqueous methanolic solution mixture (30/70%) for 3 days and filtered. The whole process was repeated thrice. To obtain a thick form of crude extract of *P. bistorta* roots, the filtrate was dried in a rotary evaporator (Heidolph, Germany) at a lower temperature and pressure and named *Pb.Cr,* which was stored at −20 °C to utilize in further experimental procedures.

### 2.2 Animals

Male Sprague Dawley rats (180–220 g) and Wistar Albino mice (25–35 g) were acquired from the animal house of the Pharmacology Department, IUB, Pakistan’s Animal House, Reference No. PAEC/24/111. The rats were housed in ventilated cages with six rats per cage, in a controlled environment at 24 °C ± 4 °C and 60% humidity, and a 12–12 h light–dark cycle. The animals included in the experiment had frequent access to food and water. The Pharmacy Animal Ethics Committee (PAEC), IUB, authorized every experimental protocol used in the study. Before beginning the trial, the animals were given 15 days to acclimatize.

### 2.3 Chemicals

LPS: Cat. # (4391) and PBS: Cat. # (4474) were purchased from Sigma-Aldrich. ELISA Kits: SOD- ELK (5616), MDA-ELK (8612), and AChE-ELK (5988) were purchased from ELK Biotechnology Co., Ltd. For oxidant/antioxidant status, TAC-615700–1 and TOS-IKJU-002CL assay kits were used. RNA kits and the Advantage RT-for-PCR for cDNA application were obtained from Thermo Fischer Scientific, United States. All chemicals and reagents used, such as normal saline, methanol, Folin–Ciocalteu reagent, sodium carbonate, distilled water, ketamine, xylazine, eosin, hematoxylin, potassium dihydrogen sulfate, wright and Giemsa stains, paraffin, formalin, aluminum chloride, sodium nitrate, and dexamethasone, were analytical grade.

### 2.4 *In vitro* studies

#### 2.4.1 Total phenolic contents (TPC)

*Pb.Cr* was assayed for TPC using the Folin–Ciocalteu (FC) method as per Masood et al. (2023) by minor adjustments. In brief, 1 mL of *Pb.Cr* was combined with 2.5 mL of 10% FC reagent. After 5 min, 2.0 mL of 75% Na_2_CO_3_ was included in the solution and incubated for 30 min. Using a blank, absorbance was taken at 765 nm on a Shimadzu UV-1800 spectrophotometer. The description of the data was in mg GAE/g *Pb.Cr*.

#### 2.4.2 Total flavonoid contents (TFC)

TFC was evaluated by AlCl_3_ colorimetric assay as per Masood et al. (2023). *Pb.Cr* 1 mg/mL of quercetin dilutions were mixed with 4 mL of dH_2_O in a flask. Then, 5% NaNO_2_ (0.3 mL) and 0.3 mL of 10% AlCl_3_ were added to the flask, and 2 mL of IM NaOH was added, the final volume being 10 mL. Absorbance was taken at 510 nm, whereas TFC was expressed as mg QE/g *Pb.Cr*.

#### 2.4.3 AChE (acetylcholinesterase) and BChE (butyrylcholinesterase) inhibition assay

The inhibitory activities of AChE and BChE of *Pb.Cr* were measured as per Ellman et al. (1961). The entire 100 µL reaction mixture for both inhibition experiments was prepared, consisting of 60 µL KH_2_PO_4_ buffer (100 mM, pH 7.7), 10 µL test substance (extract) (0.5 mM each well), and 10 µL enzyme, in that order. Subsequently, all contents were mixed and pre-incubated at 37 °C for 10 min, and the absorbance at 405 nm was measured. The cholinergic iodide (10 μL, 0.5 mM per well) substrate was mixed to initiate the reaction. Next, 10 µL of DTNB (0.5 mM per well) was added, and at 405 nm, absorbance was recorded. Every experiment was conducted three times in duplicate, along with the standard control (eserine). According to the formula below, a drop in absorbance value indicates increased radical scavenging activity.

$$\text{Inhibition }\left(\%\right)=\text{Control}-\text{Test }/\text{ Control}\times 100$$

The data are represented as mean ± SD (n = 3). Significant differences (p < 0.05, Tukey test) are expressed by different letters. IC_50_ = the half-maximum inhibitory concentration of AChE and BChE.

#### 2.4.4 GCMS analysis

To analyze *Pb.Cr*, GC-MS was performed (Agilent technologies USA) as per Nawaz et al. (2023). A Hewlett-Packard 5890 II GC was utilized, equipped with an HP-5 capillary column (30 m, 0.25 mm i.d., 0.25 μm film thick) and a mass spectrometer 5971 A as detector. The carrier gas, helium, flowed at an average pace of 1 mL/min. The column’s temperature increased from 160 ℃ to 240 ℃. For GC-MS detection, an electron ionization apparatus with ionization energy of 70 electron volts (eV) was utilized. The extracts were diluted 1:100 (v/v) with diethyl ether, and an automated splitless injecting 1.0 μL of the dilute specimens was performed.

### 2.5 *In vivo* studies

#### 2.5.1 Toxicity studies

Healthy albino mice (25–35 g) were used for acute toxicity studies of *Pb.Cr*. There were five groups of six animals each. The mice were treated orally according to groups: group I (normal saline, 10 mL/kg), test groups II, III, and IV (*Pb.Cr* at the doses of 0.3, 1, 3, and 7 g/kg), and V. Touch and pain response, sweating, convulsions, dizziness, urination, rightening reflex, overactivity, and temperature were observed at 1, 2, 5, 7, 24, and 48 h, then at 7 and 14 days after treatment. The animals had free access to diet and water.

#### 2.5.2 Lipopolysaccharide (LPS)-induced model of ALI

The process outlined by Hussain et al. (2019) was followed with some modifications to create the LPS-induced ALI model. We randomly assigned 36 mature male Sprague Dawley rats to six groups of six rats each: control (NS, 10 mL/kg), LPS-intoxicated group (4 mg/kg, 10µL/10 g, intratracheal), and extract treated three groups (i.e., 100, 300, and 500 mg/kg p.o. + LPS). The last group was the reference group, treated with dexamethasone (1 mg/kg p.o.) and intra-tracheal LPS. The animals’ weight and saturation level in oxygen were measured both prior to and after the entire procedure. Except for the LPS-intoxicated animals, every animal got the appropriate care as previously mentioned. Following an hour of therapy, every animal aside from the normal control was put to sleep with an administration of ketamine (50 mg/kg) and xylazine (10 mg/kg), and they were given LPS intratracheally. After 8 h, another oral dose of NS, plant extract, and dexa was given to all the animals except the LPS-treated group. The rats were subjected to SO_2_ assessment before LPS-challenge 24 hours after LPS exposure using a wrist-type pulse oximeter. Then all animals were again anesthetized to collect BALF, blood, and tissue samples.

#### 2.5.3 BALF and blood collection and cell count

The procedure for BALF collection and inflammatory cell count in BALF and blood samples was followed as per Hussain et al. (2019). The right lung was carefully collected, while the left lung was lavaged three times via syringe with 3 mL sterile normal saline (NS) intratracheally administered to obtain BALF. The NS was gently aspirated whilst rubbing each rat’s thorax to recover the solution. The process was repeated thrice and BALF collection was stored in ice containing PBS at 4 ℃.

Using a Neubauer chamber and a light microscope, an equal amount of BALF and 1% acetic acid solution were utilized to examine the inflammatory cells in the BALF sample. This process was employed to evaluate the established model’s degree of toxicity. The leftover BALF was then centrifuged for 10 minutes at 1000 x g. The supernatant was carefully collected and set aside for later use. Pellets were developed on glass slides using sedimentary concentrated fluid, which was first stained with Wright (4–7 min) and Giemsa (10–15 min). To eliminate any remaining discoloration or solid debris, these slides were washed with normal water for 1 to 2 mins. Pellets were prepared for analysis, and 200 cells were used to confirm the morphological changes of leukocytes—neutrophils, macrophages, and lymphocytes—under a light microscope using DLC. Photomicrographs were also taken using a camera mounted to the lab microscope. Blood samples were obtained via the heart puncture method for use in subsequent procedures and tests. Blood smears of each group were prepared with the aid of Wright–Giemsa stains. Cells were also counted on blood smears to check the level of systemic toxicity due to direct toxin (LPS) in animals.

#### 2.5.4 Lung–weight ratio

The lung–weight ratio was obtained by dividing the total body weight of each rat by its individual lung weight, in accordance with the directory of respiratory edema (Wu et al., 2022).

#### 2.5.5 Histopathological evaluation of lung tissues

The lower lobe of each rat’s left lung was preserved in 10% formalin for histological examination. After being fixed in paraffin, the preserved lobes were stained with hematoxylin and eosin (H & E). Next, using a five-point scoring system, lung edema, inflammation, and inflammatory cell infiltration were assessed. The grading system was 0 = normal, 1 = extremely mild, 2 = mild, 3 = moderate, 4 = marked, and 5 = significant inflammation. At least three distinct locations were used to execute the scores for each lung (Murray et al., 2014).

### 2.6 Redox signaling tests on BALF

#### 2.6.1 TOS and TAC

LPS-induced TOS and extract-induced antioxidant capacity (TAC) were assessed by following the procedure detailed by the manufacturer of kits.

The total quantity of oxidant molecules in the sample was correlated with the color intensity, which is spectrophotometrically measurable. Hydrogen peroxide (H_2_O_2_) was used to calibrate the assay, and the findings were reported in micromolar hydrogen peroxide equivalent per liter (μmol H_2_O_2_ Eq/L).

A semi-auto biochemistry analyzer (Biosystem BTS-330) was used for spectrophotometric assessment. A monochromatic wavelength of 660 nm was selected for the procedure. We added 200 μL of acetate buffer had with 5 µL of sera or standards. The mixture was then given 20 µL of ABTS. Trolox, as a calibrator in the identification of the TAC values, was used. The results obtained are represented in mmol Trolox equiv./L.

#### 2.6.2 MDA and SOD

SOD and MDA assay kits were used to measure the two enzymes as per the guidelines. Antioxidant enzyme activities were demonstrated by the SOD content, and lipid peroxidation was indicated by the amount of MDA present.

In brief, 200 μL of working solution was mixed with 20 μL of each extract solution at a concentration of 5 mg/mL, and the mixture was then incubated for 20 min at 37 ℃. Butylated hydroxyanisole (BHA) at a concentration of 5 mg/mL was one of the positive controls. Blank 1 comprised 200 μL of working solution and 20 μL of enzyme working solution, including 20 μL of distilled water (dH_2_O). Blank 2 comprised 200 μL of working solution, dilution buffer, and 20 μL of each plant extract. Blank 3 was created similarly to blank 2, with the exception that 20 μL of dH_2_O was substituted with the plant extract. The SOD activity was ultimately calculated after the mixture’s absorbance at 450 nm was measured.

MDA concentration in blood serum was estimated using a thiobarbituric acid (TBA) test. This test is based on the reaction between MDA and TBA, resulting in the formation of red color adduct (secondary by-product), measured using a spectrophotometer. The serum samples (0.5 mL) were mixed with 2.5 mL of 20% trichloroacetic acid and 1 mL of 0.67% TBA and then vortexed. For half an hour, the mixture was heated to 100 ℃ in a water bath. After cooling, 4 mL of n-butanol was added to each tube, and they were both centrifuged for 10 min at 2000 g.

Melondialdehyde (MDA) = absorbance/0.94–0.1322 was the result of measuring the absorbance at 532 nm after the supernatant was removed.

### 2.7 qRT-PCR

Lung sample RNA was isolated using a TRIzol (ThermoFisher Scientific USA) mixture. Following isolation, RNA samples were quantified using the Nanodrop, Thermo Scientific Multiskan Go, Rev. 1.3, Cat No. N10588. Using the cDNA synthesis kit from ThermoFisher Scientific, cDNA was synthesized from 1 g of RNA in each of the samples as per manufacturer’s instructions.

qRT-PCR was performed on the iQ5 Bio-Rad using the Maxima SYBR Green/ROX Master Blend. The beta-actin gene was the housekeeping gene. For each of the above genes, the cDNA was denatured for 15 s at 95 ℃ using the following PCR method. For a total of 39 cycles, the annealing temperature was initially set at 58 ℃ for 30 s. It was then extended for 20 s at 72 ℃. Ten seconds at 95 ℃ and 0.2 ℃ increments between 62 ℃ and 95 ℃ were used in the melt curve methodology. Data collection was made possible at every melt curve increment. Melt and standard curves were created by the CFX manager software (Version 2.0). Lastly, the qRT-PCR data were analyzed using the 2*(-ct) method. The fold change expression of the targeted gene was computed using these values.

The primers utilized in every experiment are listed in Table 1 below.

**TABLE 1:** List of primers for qRT-PCR Sequencing.

Gene	Primers 5′-3′
***IL-6***	F: CCACCCACAACAGACCAGGTA R: CGGAACTCCAGAAGACCAGAG
***IL-1β***	F: CACCTCTCAAGCAGAGCACA R: ACGGGTTCCATGGTGAAGTC
***COX-2***	F: GCGGGTGACTAGAAGGTCC R: GAATGTGGCGGCTCCCAAC
***NF-ĸβ***	F: AAGATGTGGTGGAGGACCTT R: GGTGGTTGATAAGGAGTGCT
***TNF-α***	F: GCCTCTTCTCATTCCTGCTTG R: CTGATGAGAGGGAGGCCATT
***β-Actin***	F: GGCTATAGTCACCTCGGGGC R: GTAATAATGCGGCCGGTCTG

F represents forward, and R indicates reverse.

### 2.8 Molecular docking

The 3D crystal structures of acetylcholinesterase were taken from the Protein Data Bank (PDB: 63 kDa, R: 2.50 Å) (https://www.rcsb.org/, accessed on 3rd JAN, 2024) to proceed with the molecular docking procedure. After adding the polar hydrogens and removing the HETATM, water, and other extra chains, the PDB file was changed into PDBQT format. PUB Chem ((https://pubchem.ncbi.nlm.nih.gov/, accessed on 3rd JAN, 2024) was used to obtain ligands to evaluate their potential against the prepared protein molecules. PyRx 0.9.x., a virtual screening software, was used to minimize all the energy of the ligands once they had been loaded into Open Babel. The PDBQT format molecules were then loaded to conduct additional research. AutoDock Vina and Vina Wizard were used to perform autodock of all the selected molecules. The pattern box (24 × 24 × 24) was then created with precise measurements. The docked molecules were saved into an Excel file. All types of interactions among structural conformations were analyzed *via* the BIOVIA Discovery Studio 2021.

### 2.9 Statistics

Data were expressed as mean ± SEM. For statistical significance between the test, standard, and intoxicated groups, one-way analysis of variance (ANOVA) with Tukey’s test was applied to each pair of columns in the acquired data using GraphPad Prism software (8.0.2). *p* > 0.05 was regarded as non-significant (ns), *p* < 0.05 as significant (^*^), *p* < 0.01 as more significant (^**^), and *p* < 0.001 as extremely significant (^***^/^###^).

## 3 Results

### 3.1 *In vitro* research

#### 3.1.1 TPC and TFC

TPC of *Pb.Cr* was calculated by the standard curve of gallic acid and presented as gallic acid equivalents (GAE) per gram of dry weight of the sample. The deliberate quantity of TPC in *Pb.Cr.* using a standard calibration curve (Y = 0.0052 × 0.0217, *R* ^2^ = 0.9808) was found to be 156.2 ± 5.13 GAE/g. A calibration curve of standard quercetin equivalents mg QE/g of *Pb.Cr* was used to estimate the total flavonoids. TFC in *Pb.Cr* was calculated at various concentrations ranging 10–50 mg/mL curve (Y = 0.0028x + 0.1218, *R* ^2^ = 0.9516), designed to possess up to 179.45 ± 2.08 mg QE/g.

#### 3.1.2 Enzyme inhibition assay

IC_50_ values for AChE and BChE may vary, with AChE sometimes showing slightly higher sensitivity to eserine. Inhibition percentages can also differ based on the enzyme’s activity level, substrate type, and assay conditions. The results revealed that *Pb.Cr* at a 15 mg/kg dose has significant acetylcholine esterase inhibition activity, and the results are comparable to standard eserine, while the extract had insignificant BChE inhibitory activity.

#### 3.1.3 Gas chromatography–mass spectrometry (GC–MS) analysis

**FIGURE 1:**
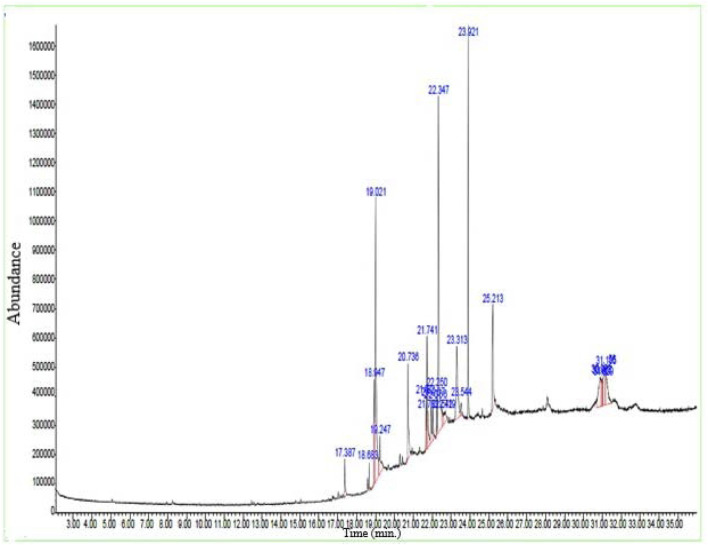
GC –MS spectra of *Pb.Cr* expressing different bioactive metabolites with peaks and retention time (RT).

By using the GC–MS technique, it was possible to identify plant metabolites with known pharmacological potential (Table 3; Figure 1). The GC–MS profile of *Pb.Cr* showed the presence of 24 metabolites: n-hexadecanoic acid (17.38%), cis/trans-13-octadecanoic acid methyl ester (18.68%), 10E-12Z-octadecadienoic acid (18.94%), oleic acid (19.02%), cis-vaccenic acid (19.24%), 13-eicosenoic acid (20.73%), 7-pentadecyne (21.68%), 9-octadecenoic acid (Z)-, oxiranylmethyl ester (21.74%), cyclopentadecanone, 2-hydroxy- (21.78%), pyridine-3-carboxamide, oxime, N-(2-trifluoromethylphenyl)- (21.96%), 13-docosenoic acid, methyl ester (22.06%), phthalic acid, di (2-propylpentyl) ester (22.25%), 18-nonadecenoic acid (22.34%), erucic acid (22.54%), cyclopentadecanone, 2-hydroxy- (22.71%), (R)-(−)-14-methyl-8-hexadecyn-1-ol (23.31%), 1,2-benzisothiazol-3-amine (23.54%), 1,4-benzenedicarboxylic acid (23.92%), cyclohexanecarboxylic acid (25.21%), 6-octadecenoic acid, (Z)- (30.90%), 1,2-benzenediol, 3,5-bis(1,1- dimethylethyl)- (31.02%), n-propyl 11-octadecenoate (31.15%), and i-propyl 9-octadecenoate (31.18%).

### 3.2 *In vivo* research

#### 3.2.1 Acute toxicity study

An acute toxicity study was conducted to estimate the lethal dose (LD_50_). *Pb.Cr* was administered in mice at single doses of 0.3, 1, 3, and 7 g/kg by the animal’s weight p.o. and was continuously observed for the initial 1, 2, and 12 hours, and then for a further 72 h to identify any harmful consequences that might have occurred after the treatment time. For 14 days, all animals were under constant observation. The lethal dose (LD_50_) for this plant falls beyond the highest dose of 7 g/kg. Macroscopic examination monitored all signs of behavior: respiration, light reflex, corneal reflex, eye color, grooming, urination, gripping strength, skin color changes, rightening reflex, convulsions, or no death. There were no discernible changes in any of the groups’ numerous metrics. Thus, at a maximum dosage of 7 g/kg, this plant seemed safe.

#### 3.2.2 Effect of *Pb.Cr* treatment on LPS-induced hypoxemia and lung edema

**FIGURE 2:**
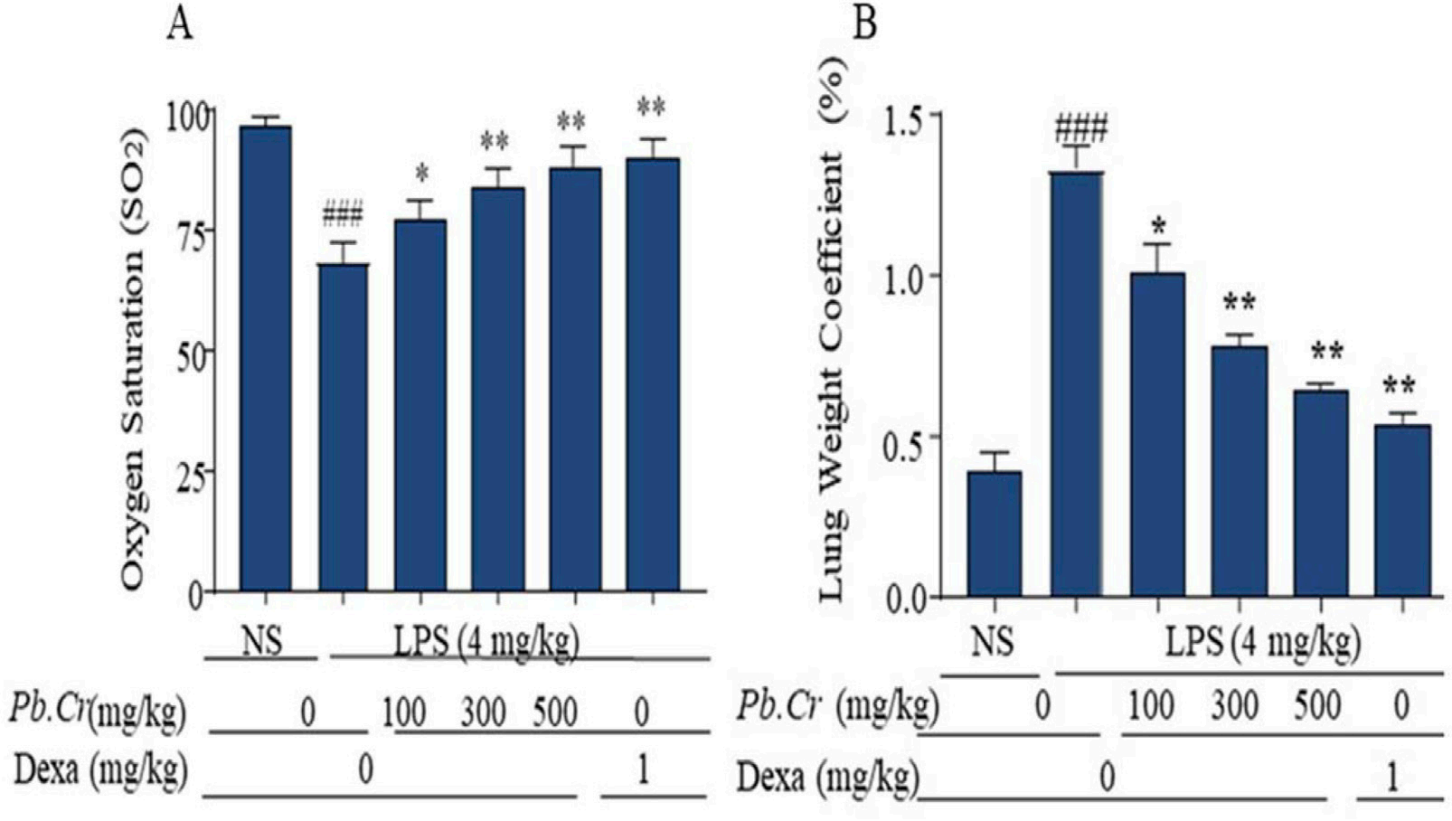
Indicating the **(A)** level of Oxygen Saturation (SO2) and **(B)** Lung Weight Coefficient (pulmonary edema) of every experimental group. SO_2_ was measured before dissection of animals. LPS (4 mg/kg i.t.) was administered to the LPS-intoxicated group. *Pb.Cr* was administered in different doses: 100, 300, and 500 mg/kg of body weight. Lung-weight coefficient was calculated by dividing the lung mass of each rat by its body weight. Tukey’s test along with one-way ANOVA was used to statistically assess the data.^*^ *p* < 0.05 and ^**^ *p* < 0.01 vs model group^; ###^ *p* < 0.01 vs. treatment groups (mean ± SEM; sample no. = 6).

*Pb.Cr* treatment expressed a significant change in hypoxemia and lung edema stimulated by LPS in experimental animals. LPS-intoxicated animals displayed more hypoxemia than control, whereas 300 and 500 mg/kg of *Pb.Cr* more significantly (*p* < 0.01) improved oxygen saturation in animals in a dose-dependent manner. Lung weight coefficient expressed LPS producing more edematous conditions in the LPS-challenged group than control. *Pb.Cr*. at 100 mg/kg also showed significant (*p* < 0.05) change in hypoxemia as well as lung edema (Figures 2A, B).

#### 3.2.3 Effect of treatment of *Pb.Cr* on LPS-induced inflammatory cell count

**FIGURE 3:**
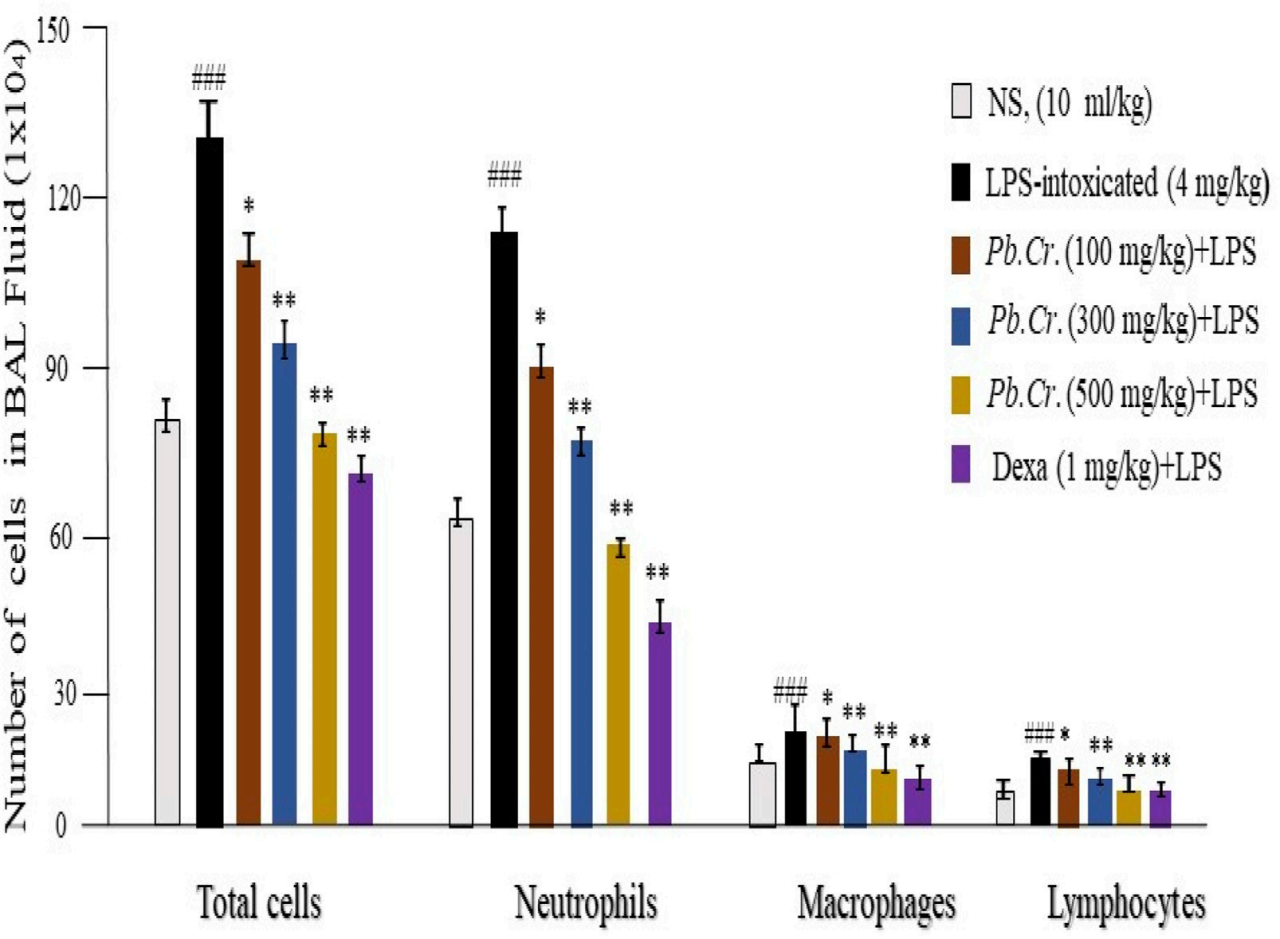
Patterns of macrophage, lymphocyte, neutrophil, and total cell infiltration in the BALF. The technique of counting cells was used to calculate the degrees of inflammation and cell infiltration. *Pb.Cr* reduced the quantity of inflammatory cells in the BALF and blood of test groups. A sufficient quantity of BALF was retrieved 24 h after the LPS ingestion, and the total number of cells in the Neubauer chamber was counted. Following the overnight dispersion of pellets obtained by BALF centrifugation on the slide, inflammatory cells, neutrophils, macrophages, and lymphocytes were identified using Wright–Giemsa staining under a light microscope based on their morphological criteria. Using a microscope, differential leukocyte counter (DLC), and Giemsa-stained pellets, 200 cells were counted. Monocytes have kidney-shaped axes, while neutrophils have two to five projection nuclei when fully formed. Graphical explanations of the inflammatory cell count. ^**^ and ^*^ *p* < 0.01 in comparison to the treatment groups; ^###^ in comparison to the control group (mean ± SEM; n = 6).

LPS-induced inflammatory cell count was executed *via* BALF after centrifugation. The 200 cells were counted on pellets of BAL fluid from each group of the ALI model. Cell counting through the blood samples of each group also indicated more toxicity and inflammatory conditions in rgw intoxicated group than in the Pb.Cr, dexa, and control groups. The LPS-treated group expressed a large amount of inflammatory cell infiltration—neutrophils, macrophages, and lymphocytes—than the normal control group. The dexa-treated group showed similarity to the control group, whereas the *Pb.Cr*-treated groups revealed less infiltration of inflammatory cells than the LPS-intoxicated animals (Figure 3). Transmigrated neutrophils, activated macrophages, lymphocytes, and total cells were taken into account, and the findings showed an almost three- to four-fold elevation in the penetration of inflammatory cells, mainly neutrophils, in LPS-treated animals. *Pb.Cr* defended against LPS-induced ALI (Figure 3).

#### 3.2.4 Effect of treatment of *Pb.Cr* on lung histopathology

**FIGURE 4:**
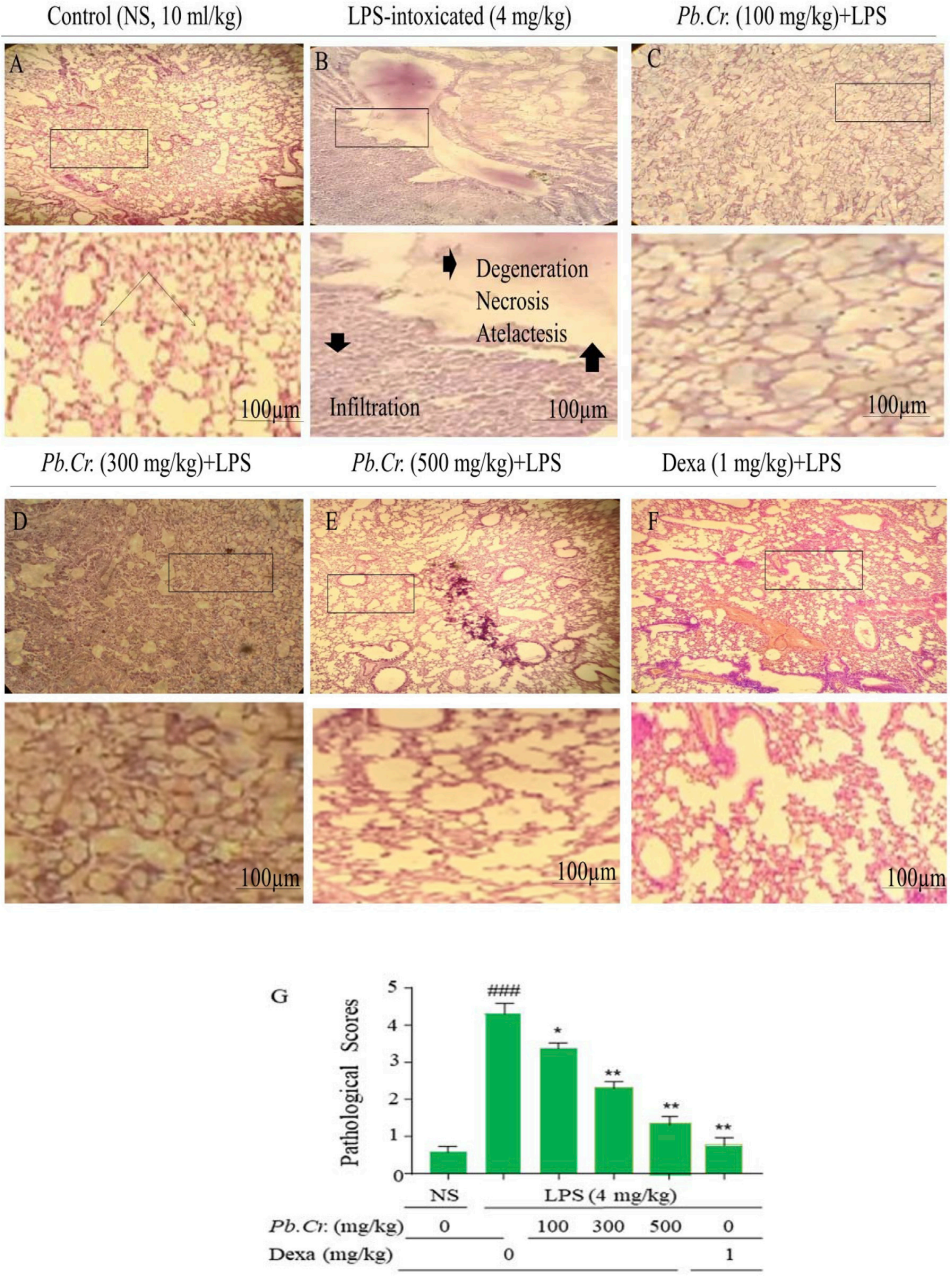
Figure indicating the effects of pre- and post-treatment of *Pb.Cr* on histology of lung tissues dyed with H & E in control **(A)**, LPS-intoxicated **(B)**, *Pb.Cr* treated (100, 300, and 500 (mg/kg, **(C–E)**), and dexamethasone (1 mg/kg) **(F),** while **(G)** describes the pathology scores that account for LPS intoxication, followed by the treatments’ levels of recovery with *Pb.Cr*. Following the euthanizing of every animal, the lower lobes of the right lung were removed, cleaned in saline, and preserved in 10% formalin for a full day. After the tissues were encased in paraffin and cut into 4–5 µm pieces using a microtome, slides were made with H & E stains such that, by adjusting optical diameters, a light microscope could see the histology of the lungs in pathologic *versus* normal conditions where ^*^ *p* < 0.5 is taken as significant, ^**^ *p* ≤ 0.01 taken as more significant, and *p* < 0.05 and ^**^ *p* < 0.01 vs treatment groups; ^###^ *p* < 0.01 vs control group with mean ± SEM, sample no. = **6**.

Histopathological investigation expressed normal architecture in the control group (Figure 4A). LPS-intoxicated animals showed extreme degeneration of epithelial, alveolitis, infiltrated inflammatory cells or margination, pneumonia, and atelectasis in LPS-intoxicated animals (Figure 4B) vs control, whereas a decline in lung structure impairment at pre- and post-treatment of *Pb.Cr* and dexa in a dose-dependent way compared to the intoxicated group was observed. *Pb.Cr* 100 mg/kg expressed significantly less pathological condition than the LPS-intoxicated group (Figure 4C), and 300 and 500 mg/kg of *Pb.Cr* showed a marked recovery in damaged lung (Figures 4D, E). Histopathological findings described problems such as cell infiltration, pneumonia, atelectasis, bronchitis, pulmonary edema, and vascular degeneration in LPS-treated animals, while 300 and 500 mg/kg *Pb.Cr* markedly reduced the severity of complications. No histological alterations were noted in the control group compared to the LPS-intoxicated group (Figure 4). The decision was made to produce inflammatory scores by applying Murray’s scoring system.

#### 3.2.5 Effect of treatment of *Pb.Cr* on LPS-induced redox signaling pathways

**FIGURE 5:**
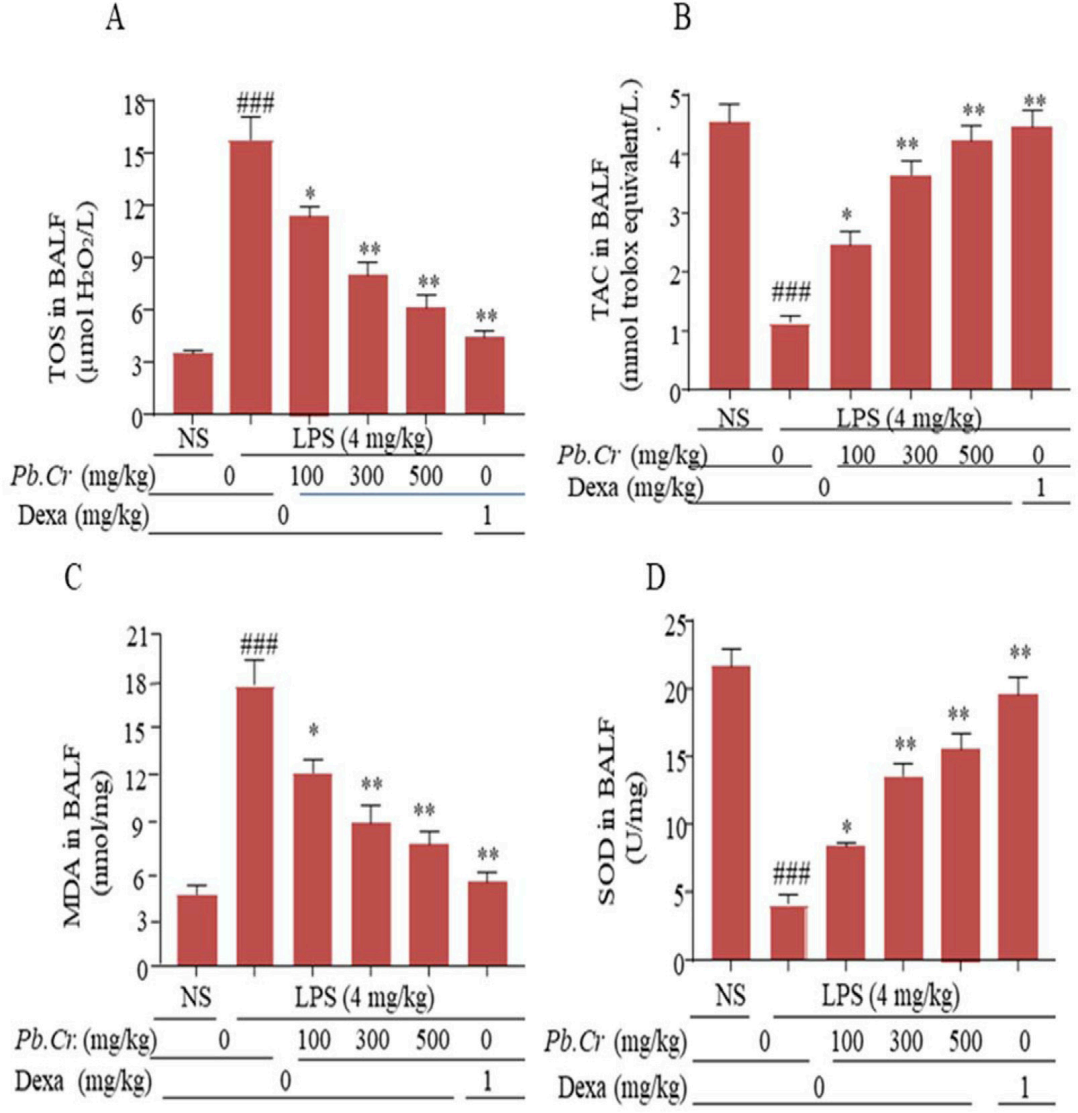
Graphical expression indicating the TOS **(A)**, TAC **(B)**, MDA **(C),** and SOD **(D)** of *Pb.Cr* in BALF of respective tissues of the LPS-induced ALI murine model. Graph represents the effects of pre- and post-treatment of *Pb.Cr* (100, 300, and 500 mg/kg) and dexa on the LPS-intoxicated ALI murine model. ^*^ *p* < 0.01 taken as more significant, ^##^ *p <* 0.01 vs control group; ^*^ *p <* 0.05 and ^**^ *p <* 0.01 vs model group with mean ± SEM; sample no. = 6).

**FIGURE 6:**
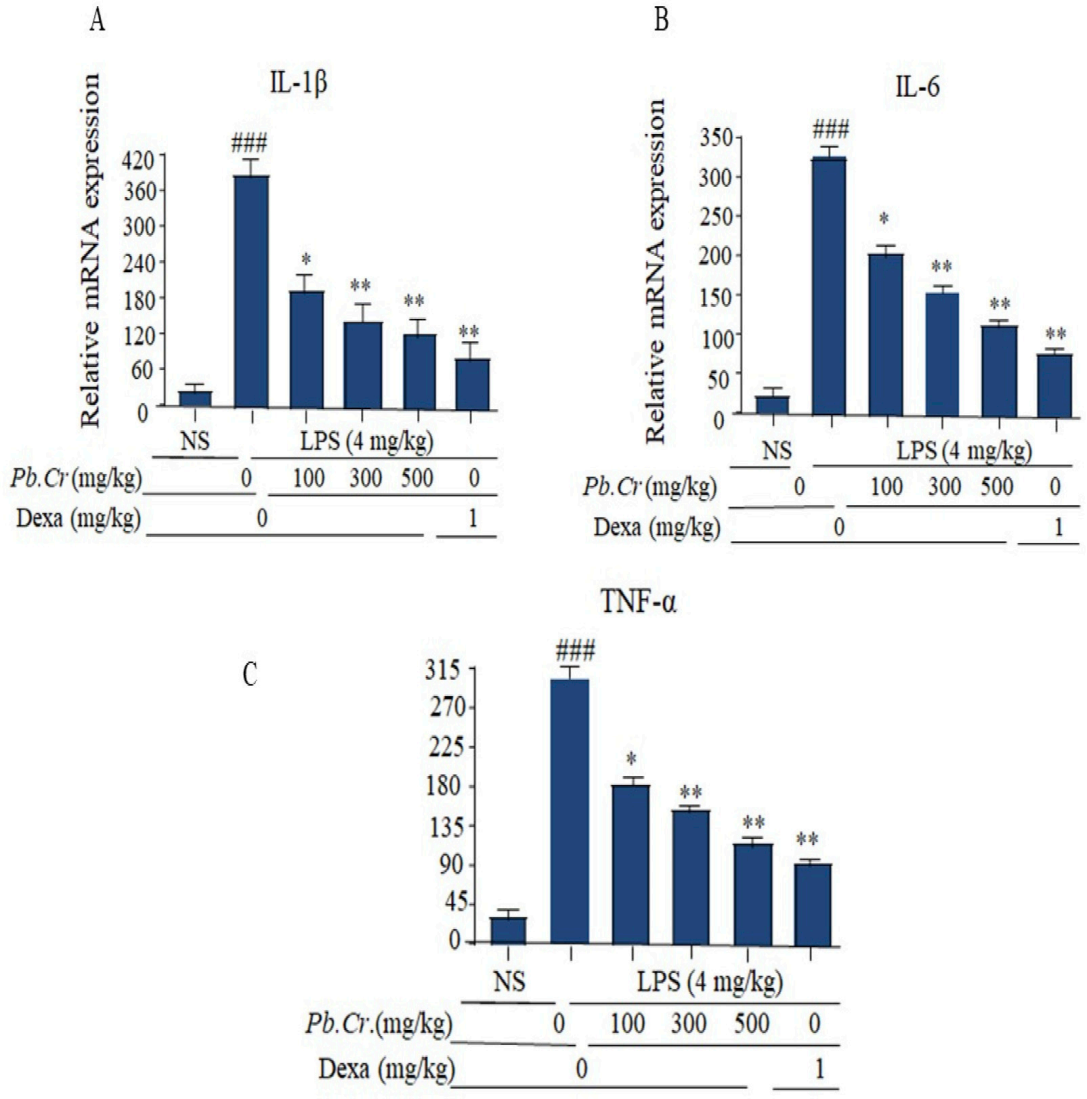
Figure showing gene expression levels of IL-1β, IL-6, and TNF-α (fold change) in order to evaluate acute damage to the lungs in the experimental model. **(A)** Level of relative mRNA of IL-1β in the tissues of the LPS-induced ALI murine model. **(B)** Level of IL-6 in the tissue samples of the LPS-induced ALI murine model and **(C)** relative mRNA amount of TNF-α in corresponding tissues of the LPS-induced ALI murine model. The effects of treating an LPS-intoxicated ALI murine model with *Pb.Cr.* (100, 300, and 500 mg/kg p.o. plus LPS) and dexa (1 mg/kg p.o. plus LPS) are shown in the graph. ^*^ *p* < 0.01 taken as more significant, ^###^ *p <* 0.01 vs control group; ^*^ *p <* 0.05 and ^**^ *p <* 0.01 vs treatment groups*.* With mean ± SEM; sample no. = 6).

ELISA kits were employed to assess the levels of TOS, TAC, MDA, and SOD. Pre- and post-administration of *Pb.Cr* at three doses (100, 300, and 500 mg/kg) improved the levels of redox signaling pathways—TOS, TAC, SOD, MDA—`in all the experimental groups, whereas the LPS-intoxicated group showed an increase in oxidation (TOS) and MDA values and a decline in the anti-oxidant capacity and super-oxide dismutase levels in intoxicated animals (Figure 5). All the doses of *Pb.Cr* reduced MDA, and TOS levels were increased via LPS-induced oxidative damage in the airways. On the contrary, the levels of SOD and TAC were amplified with *Pb.Cr* administration (Figure 6).

#### 3.2.6 Effect of treatment of *Pb.Cr* on LPS-induced IL-1β, IL-6, and TNF-α

Pro-inflammatory cytokines, such as IL-1β, IL-6, and TNF-α (fold change), were considerably more escalated in the LPS-intoxicated animals than control. The doses of 300 and 500 mg/kg *Pb.Cr* ameliorated more significantly (*p* < 0.01) than LPS-intoxicated and standard groups. (Figures 6A–C). PCR analysis showed increased levels of pro-inflammatory cytokines—IL-1β, IL-6, and TNF-α—in the LPS-intoxicated group, while *Pb.Cr (*300 and 500 mg/kg) ameliorated the cytokines (Figures 6A–C).

#### 3.2.7 Effect of *Pb.Cr* treatment on LPS-induced COX-2

**FIGURE 7:**
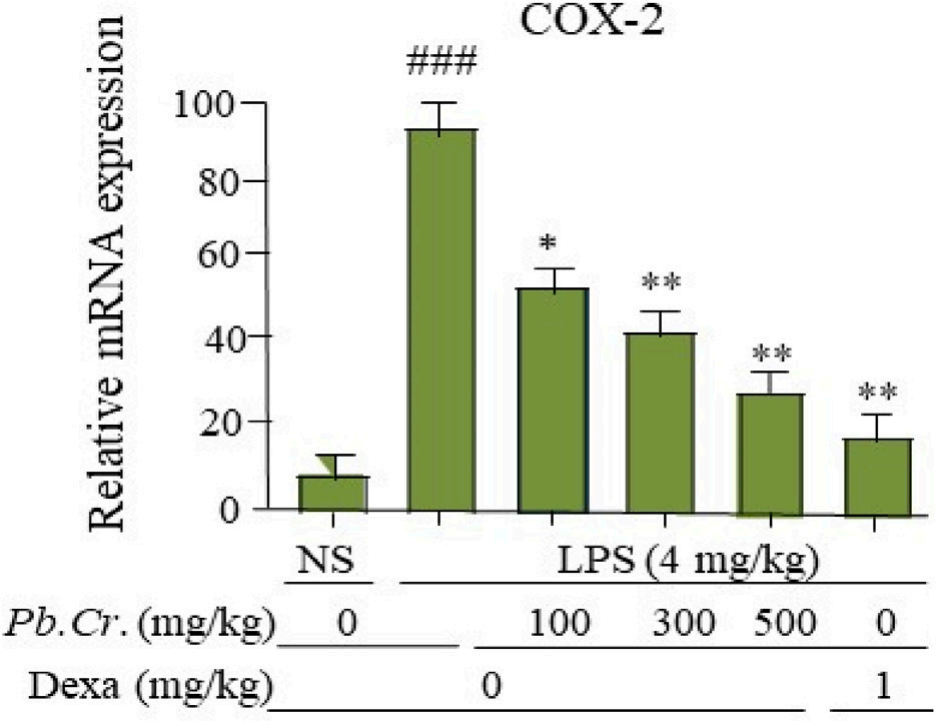
Figure indicating the mRNA levels (change) of COX-2 in the respective tissue samples to evaluate the lung protection by *Pb.Cr* and dexamethasone in the LPS-induced ALI murine model. Graph shows the marked effects of different doses of plant extract with pre- and post-treatment (100, 300, and 500 mg/kg) and dexa on the LPS-intoxicated ALI murine model. All readings were measured thrice for each of sample. ^*^ *p* < 0.01 taken as more significant, ^##^ *p <* 0.01 vs control group; ^*^ *p <* 0.05 and ^**^ *p <* 0.01 vs. treatment groups with mean ± SEM; no. of samples = 6.

A key enzyme in the conversion of arachidonic acid to prostaglandin E_2_ is cyclooxygenase-2 (COX-2). The two doses (300 and 500 mg/kg) of *Pb.Cr* reduced COX-2 levels significantly (*p* < 0.01) more than the LPS-intoxicated rats with significantly enhanced levels of COX-2, whereas 100 mg/kg of *Pb.Cr.* reduced significantly (*p* < 0.05) (Figure 7). The level of COX-2 was more increased in the LPS-treated group than control while significantly lowered in *Pb.Cr.*-treated animals (Figure 7).

#### 3.2.8 Effect of treatment of *Pb.Cr* on LPS-induced NF-ĸβ

**FIGURE 8:**
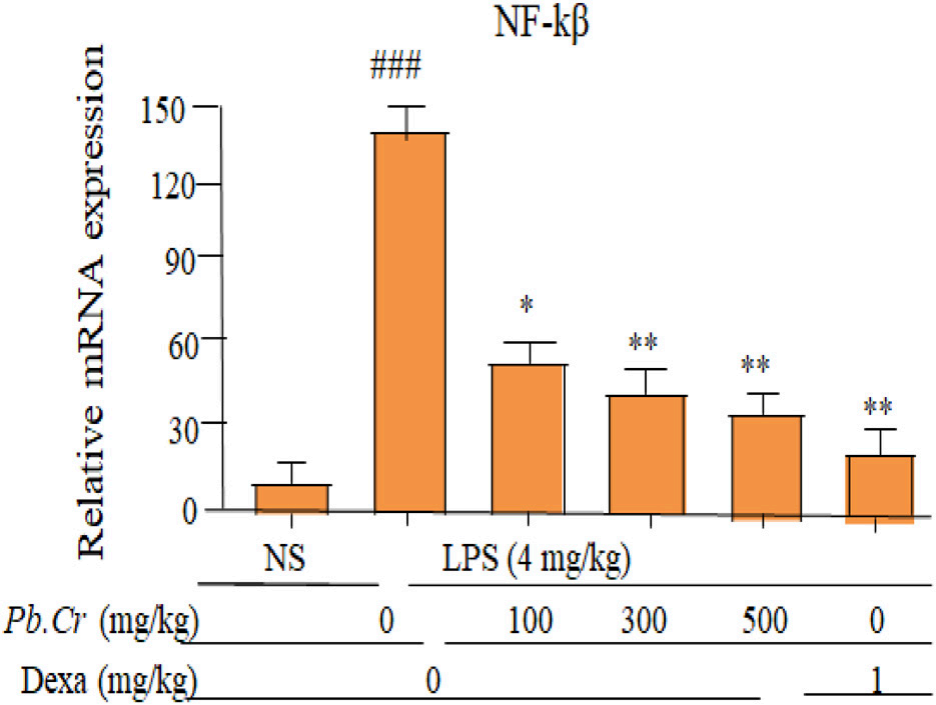
Graph indicating levels of mRNA of NF-ĸβ (fold change) of *Pb.Cr* in the lungs of respective animals of LPS-induced ALI in rats. Graph shows the toxic effects of LPS and ameliorative effects of *Pb.Cr* with pre- and post-treatment (100, 300 & 500 mg/kg) and dexa on the LPS-intoxicated ALI murine model. ^*^ *p* < 0.01 taken as more significant, ^##^ *p <* 0.01 vs control group; ^*^ *p <* 0.05 and ^**^ *p <* 0.01 vs treatment group (mean ± SEM; n = 6).

*Pb.Cr* at 300 and 500 mg/kg improved the levels of NF-ĸβ more significantly (*p* < 0.01) than in LPS-intoxicated animals whereas 100 mg/kg of *Pb.Cr.* reduced significantly (*p* < 0.05) in animals (Figure 8).The stimulation of NF-ĸβ was enhanced in the LPS-intoxicated group than control, and declined levels of NF-ĸβ were observed in all the animals treated with *Pb.Cr* (Figure 8).

### 3.3 Molecular docking

Molecular docking is carried out on three fundamental criteria: bond characterization, molecular connections, and bond intensity. Some metabolites have a good ADME profile, hydrogen bonds, van der Waals interactions, and relatively tiny bond energies (Prasanth et al., 2021). Eight metabolites were thus selected from the GC–MS profile of *Pb.Cr* and docked against choline esterase to link them to the test enzyme inhibition results and obtain a deeper comprehension of the metabolites’ prevention capacity. The docked metabolites with maximum binding affinities—phthalic acid (−6.1), 1,2-benzenediol (−5.8), 1,2-benzisothiazol-3-amine (−7.8), 10E,12Z-octadecadienoic acid (−8.0), oleic acid (−7.4), CIS-vaccenic acid (_6.9), 7-pentadecyle (7.5), and cyclohexane carboxylic acid (−8.6) AChE—are shown in Supplementary Table S1 and Supplementary Figure S1.

## 4 Discussion

Botanical drugs have diverse anti-inflammatory and anti-oxidative potential and suggest a step toward novel drug discovery in pulmonological areas of science (Wanyo et al., 2024). This endeavor was intended to investigate the lung protective sound effects of *Pb.Cr* having bioactive metabolites against LPS-induced ALI in rats*. In vitro*, *in vivo,* and *in silico* studies were included to expand the broad range of evidence for a positive impact on health.

Research has shown that a variety of secondary metabolites may be employed in the management of ALI (Bae et al., 2010). The phenolics and flavonoids were quantified, indicating potential for anti-oxidative, anti-inflammatory, anti-bacterial, and anti-proliferative effects. The polyphenolic profile of *Pb.Cr.* obtained by this investigation was comparable to the results of Wang et al. (2016). The many phenolic metabolites, flavonoids in particular, have drawn interest because of their possible health advantages (Al-Khayri et al., 2022; Oluwole et al., 2022).

When there is acute lung injury, the lung cholinergic system plays a key role in reducing pulmonary inflammation (Pinheiro et al., 2021). Eserine (physostigmine) is a reversible inhibitor of ACHE and BChE. It is usually effective in the range of 0.1–10 µM. The degree of lung injury can be ascertained by measuring elevated levels of AChE and BChE in the lung lavage (Graham et al., 2006). The metabolites that decrease AChE potential can act as immunity boosters (Pohanka, 2014). *Pb.Cr* showed a marked inhibition of AChE. *Pb.Cr* has considerable effects on the immune system as well as anti-oxidation by enhancing the anti-inflammatory potential (Table 2).

**TABLE 2:** % Inhibition and IC_50_ of *Pb.Cr* and Standard Eserine.

Enzyme	AchE		BchE	
Sample Code	% Inhibition	IC_50_ µM	% Inhibition	IC_50_ µM
Eserine (15 mg/kg)	93.25 ± 1.13	0.04 ± 0.0001	93.25 ± 1.13	0.04 ± 0.0001
*Pb.Cr* (15 mg/kg)	81.77 ± 0.62	395.51 ± 0.27	28.57 ± 0.62	-

AChE and BChE present in the central and peripheral nervous systems, respectively (mean ± SEM, n = 3).

GC-MS of *Pb.Cr* was conducted to identify and quantify metabolites. The data obtained showed the presence of 24 metabolites in *Pb.Cr*. Among these, 15 metabolites having a Qual factor of more than 80 are given in Table 3. Di (2-propylpentyl) ester and hexadecanoic acid are among the metabolites that have been found to have antioxidant and anti-inflammatory properties (Kala et al., 2011). Prior research has shown the anti-inflammatory properties of octadecadienoic acid while naphthalene and 1,2 benzene dicarboxylic acid have anti-microbial potential (Uma et al., 2009). Pyridine-3-carboxamide is a DNA gyrase inhibitor in bacteria and is considered antimicrobial (Yule et al., 2014). Octadecanoic, oleic, erucic, and vaccenic acids are all anti-oxidant and anti-inflammatory and (R)-(−)-14-methyl-8-hexadecyn-1-ol has the potential to treat injuries (Mohansrinivasan et al., 2015). Molecular docking was also incorporated as a step for drug design for ALI/ARDS treatment strategy.

**TABLE 3:** GC/MS spectral details of isolated and identified peaks of *Pb.Cr.* (NIST Library-based).

Peak number	RT	Compound (s) identified	Area (%)	Mol. formula	Mol. weight (g/mol)	Qual	Pharm. activity
1	17.38	n-Hexadecanoic acid	1.54	C_16_H_32_O	256.42	99	Anti-inflammatory (Aparna et al., 2012)
2	18.68	Cis/trans-13-octadecanoic acid methyl ester	0.76	C_19_H_36_O	296.48	99	Anti-microbial (Suresh et al., 2014)
3	18.94	10E-12Z- Octadecadienoic acid	4.30	C_18_H_32_O_2_	280.42	99	Anti-bacterial (Mundt et al., 2003)
4	19.02	Oleic acid	16.77	C_18_H_34_O_2_	282.46	99	Tumoricidal (Suresh et al., 2014)
5	19.24	Cis-vaccenic acid	2.69	C_18_H_34_O_2_	282.461	93	Anti-oxidant and anti-microbial (Shawer et al., 2022)
6	20.73	13-Eicosenoic acid (paullinic acid)	4.32	C_20_H_38_O_2_	310.51	99	Anti-microbial (Kofi et al., 2009)
7	21.68	7-Pentadecyne	1.54	C_15_H_28_	208.38	87	Anti-cancer (Sianipar and Purnamaningsih, 2018)
8	21.74	9-Octadecenoic acid (Z)-, oxiranylmethyl ester	3.93	C_21_H_38_O_3_	296.49	99	Anti-bacterial and anti-oxidant (Premalatha et al., 2023)
9	21.78	Cyclopentadecanone, 2-hydroxy-	2.01	C_21_H_38_O_3_	296.49	__	Anti-oxidant (Kayat et al., 2016)
10	21.96	Pyridine-3-carboxamide, oxime, N-(2-trifluoromethylphenyl)-	1.92	C_22_H_42_O_2_	338.6	__	Anti-microbial and anti-oxidant (Chandra and Gunasekaran, 2017)
11	22.06	13-Docosenoic acid, methyl ester	1.31	C_6_H_8_N_2_O_2_	140.14	91	Anti-bacterial (Moni et al., 2021)
12	22.25	Phthalic acid, di (2-propylpentyl) ester	1.07	C_24_H_38_O_4_	390.6	__	Anti-cancer, Anti-microbial (Shaheed et al., 2019)
13	22.34	18-Nonadecenoic acid	15.58	C_22_H_42_O_2_		99	Anti-inflammatory (Ramya et al., 2015)
14	22.54	Erucic acid	0.90	C_22_H_42_O_2_	338.6	99	Anti-inflammatory, Anti-microbial, Neuroprotective (Galanty et al., 2023)
15	22.71	Cyclopentadecanone, 2-hydroxy-	1.48	C_22_H_42_O_2_		99	Anti-oxidant (Kayat et al., 2016)
16	23.31	(R)-(−)-14-Methyl-8-hexadecyn-1-ol	6.11	C_17_H_32_O	252.45	90	Anti-diabetic, Anti-oxidant (Sulaimon et al., 2020)
17	23.54	1,2-Benzisothiazol-3-amine	0.62	C_7_H_6_N_2_S	150.20	__	Anti-bacterial (Viani et al., 2017)
18	23.92	1,4-Benzenedicarboxylic acid	13.08	C_8_H_6_O_4_	166.13	94	Anti-proliferative (Boshra et al., 2023)
19	25.21	Cyclohexanecarboxylic acid	5.39	C_7_H_12_O_2_	128.171	__	Anti-convulsant (Liu and Pollack, 1994)
20	30.90	6-Octadecenoic acid, (Z)-	4.23	C_18_H_34_O_2_	282.46	__	n/f
22	31.02	1,2-Benzenediol, 3,5-bis(1,1-dimethylethyl)-	0.66	C_21_H_40_O	324.54	84	n/f
23	31.15	n-Propyl 11-octadecenoate	3.56	C_14_H_22_O_2_	222.32	__	n/f
24	31.18	i-propyl 9-octadecenoate	4.35	C_26_H_40_O_3_	296.49	__	n/f

RT, retention time; Pharm. Activity, pharmacological activity.

An acute toxicity study was also conducted to estimate lethal dose (LD_50_). *Pb.Cr.* was administered in mice at single doses of 0.3, 1, 3 and 7 g/kg according to the animal’s weight and was continuously observed for the initial 1, 2, and 12 hours, and then for a further 72 h to identify any harmful consequences that might have occurred after the treatment time. For 14 days, all animals were under constant observation. There were no discernible changes in any of the numerous metrics of the groups. Thus, at a maximum dosage of 7 g/kg, this plant seemed safe. A group of scientists reported no cytotoxic activity of chloroform and hexane extracts of bistorta at 100 μg/mL against cancer cell lines (Karuppiah Pillai et al., 2007). (Karuppiah Pillai , 2021) reported LD50 values of hexane and chloroform fractions of bistorta rhizomes extract in mice, inconsistent with our study.

The lung weight coefficient showed the grade of lung edema in broncho-alveolar areas. Alveolar edema, which is caused by a significant rise in the permeability of the pulmonary epithelial barrier, is one of the primary causes of hypoxemia in patients with acute ALI/ARDS. The impaired alveolar epithelium is believed to be the primary cause of amplified pulmonary permeability, which results in edema fluid having great levels of extravagated metabolites in the alveoli (Kushimoto et al., 2012). Pre- and post-treatment of *Pb.Cr.* at 100, 300, and 500 mg/kg doses expressed a significant decline in the edematous status of the pulmonary structure (Figure 2). This revealed the potential of *P. bistorta* roots against edema and inflamed states of pulmonary system by improving oxygen saturation and fluid balance.

BALF is recommended as an effective tool for describing inflammatory cellular metabolites and employs pulmonary scarring to illustrate cellular interactions in the lower respiratory system (Watters et al., 1987) and is more reliable than blood samples (Ye et al., 2021). Blood cell count was also assessed to evaluate the level of toxicity in systemic circulation. Neutrophils are an essential part of the inflammation that typifies ALI. When endotoxemia causes ALI, pro-inflammatory cytokines such as IL-1β and TNF-α are expressed by the neutrophils that migrate into the airways and infiltrate the lungs. These cytokines may also play a role in the oxidant-induced harm and degradation of epithelial strength that occur after endotoxemia (Abraham, 2003). Transmigrated neutrophils, activated macrophages, lymphocytes, and total cells were taken into account, and the findings showed an almost three- to four-fold elevation in the penetration of inflammatory cells, mainly neutrophils, in the LPS-treated animals. *Pb.Cr*. defended against LPS-induced ALI (Figure 3).

The histopathological findings described problems such as cell infiltration, pneumonia, atelectasis, bronchitis, pulmonary edema, and vascular degeneration in LPS-treated animals, while 300 and 500 mg/kg *Pb.Cr.* markedly reduced the severity of complications. No histological alterations were noted in the control group compared to the LPS-intoxicated group (Figure 4). The decision was made to produce inflammatory scores by applying Murray’s scoring system. A score of 0 indicated no lung injury, a grade of 1–2 indicated slight to moderate airway damage, a level of 3 or above suggested significant inflammation, and a level of 4–5 indicated serious harm (Raghavendran and Napolitano, 2011). Furthermore, all the doses of *Pb.Cr* declined MDA and TOS levels that were increased via LPS-induced oxidative damage in the airway. On the contrary, the levels of SOD and TAC were amplified with the administration of *Pb.Cr* (Figure 6). A lack of balance in the anti-oxidants and oxidative damage may lead to increased oxidative stress and pulmonary immune system activation (Tang et al., 2021).

PCR analysis showed increased levels of the pro-inflammatory cytokines IL-1β, IL-6, and TNF-α in the LPS-intoxicated group, while *Pb.Cr* (300 and 500 mg/kg) ameliorated the cytokines. IL-1β has become well-known for leading the inflammatory cytokine cascade (Goodman et al., 2003). The TNF-α pathway controls both cell proliferation and apoptosis at the same time, acting as a “sword with two blades”. During the phase of acute damage to tissue, irregular TNF-α signaling can set off cytokine storms that result in significant cell death. Conversely, tissue healing after the acute damage phase necessitates an ideal level of TNF-α signaling. Additionally, IL-6 is a crucial pro-inflammatory cytokine that potently induces acute inflammatory damage (Figures 6A–C). The level of COX-2 was increased in the LPS-treated group compared to control while significantly lowered in *Pb.Cr.*-treated animals (Figure 7). COX-2 inhibitors reduce proliferation as well as apoptosis in cellular levels (Mohseni et al., 2004).

NF-ĸβ stimulation was more enhanced in the LPS-intoxicated group than control, and declined levels of NF-ĸβ were observed in all the animals treated with *Pb.Cr* (Figure 8). The transcription of genes related to inflammation is started by the nuclear transcription factor NF-ĸβ. Toll-like receptors (TLR_4_) initiate NF-ĸβ via many signaling pathways (Chen et al., 2019). According to earlier studies, TLR_4_ that has been activated by LPS transmits downstream signaling, mainly through the NF-ĸβ and MAPK pathways, which in turn contributes to inflammatory reactions. (Chi et al., 2013). The five NF-ĸβ family members are NF-ĸβ_1_ (p50), NF-ĸβ_2_ (p52), Rel A (p65), Rel B, and c-Rel, which can control the transcription and translational activities of pro-inflammatory genes which are essential for controlling the inflammatory response (Alharbi et al., 2021). The suppressive protein IκBα typically sequesters the NF-ĸβ proteins in the cytoplasm. Following LPS stimulation, activated IKK causes a cascade of activations that leads to IκBα activation and depletion, which in turn releases p65. Then, phosphorylated p65 quickly moves into the nucleus and attaches to desired genes to encourage the transcription of subsequent genes (Tang et al., 2021). Here, octadecadienoic acid may decreased the amounts of phosphorylated IKKβ, IκBα, and p65 protein in a dose-dependent fashion, which may significantly inhibit p65 entering the nucleus.

Nowadays, a medication development process frequently uses computational molecular docking (Tabassum et al., 2022), which is valid for predict biochemical activities (Piccagli et al., 2008) to develop the blocker(s) of NF-ĸβ. Docking offers the benefit of detecting the type of contact between the study metabolites at the enzyme or receptor regions through particular significant relationships in addition to protein interacting structures—in this example, using chemical data from GCMS (Zhang et al., 2018). Molecular docking investigation was accomplished for AChE. A total of 23 metabolites were docked against AChE (PDB). The eight identified metabolites have the best binding affinities with this protein molecule (Supplementary Figure S1). These molecules thus have the potency to alter the molecular structures that may exert biological effects. The structural conformational changes indicated that 1,2-benzisothiazol-3-amine (−7.8) had pi-alkyl and alkyl type interactions for proline A:232 and A:529 and histidine 406, respectively, while H-bond-type interactions were observed for histidine A:390 and asparagine A:525. In interactions between proteins and ligands as well as other hydrophobic interactions like alkyl and pi alkyl, the hydrogen bond is crucial for maintaining the consistent binding of ligands to proteins (Borges et al., 2018). 10E,12Z-octadecadienoic acid (−8.0) showed the presence of H-bond and alkyl as well as Van der Waal’s force-type interactions between amino acids with different distances for each of them. For oleic acid (−7.4), H-bond-type interaction was noted for tyrosine A:121 and asparagine A:85. Pi-alkyl-type interactions were observed for five amino acids—tryptophan A:84, histidine A:440, and phenylalanine A:290, A:330, and A:331—with varying distances for each amino acid. Phthalates with binding affinity of −6.1 showed hydrophobic alkyl type interactions for valine A:323 distance of 3.23 Å, pi-sigma type interactions for valine A: 400, unfavorable Doner–Doner interactions for glutamic acid A:327 and histidine 326, and conventional H-bond type interaction for aspartic acid A:326, glycine A:441, and asparagine A:324. In CIS-vaccenic acid (_6.9), pi-alkyl-type interactions were observed for tryptophan A:85, phenylalanine A:330 and A:331, and histidine A:440 with different distance variations. Van der Waal’s force-type interactions were observed for the remaining amino acids. H-bond and alkyl type interactions were found for 7-pentadecyle (7.5). Cyclohexane carboxylic acid (−8.6) showed C-H bond, H-bond, Pi-Pi-T-shaped, and Pi-Pi-stacked type interactions between amino acids of this molecule. A major component of molecular docking is hydrogen bonding—important drug–target interactions that stabilize ligand–receptor protein complexes, leading to inhibition of microbial growth and exaggerated anti-inflammatory potential (Rozas, 2007). They can direct the ligand into the ideal binding shape and are among the strongest non-covalent interactions. One hydrogen atom can make a hydrogen bond with another electronegative atom on a different molecule if it is previously connected to a highly electronegative atom, such as oxygen or nitrogen. The binding force between a hydrogen atom and the single pair of electrons on the electronegative atom causes the hydrogen bond to form (Al-Malki, 2024). Alkyl, hydroxyl bond, Van der Waal’s forces, and covalent bonds are vital bonds that affect the short or permanent binding of ligand-receptor complexes. The binding energies are > −6.0 and the effectiveness of metabolites are strongly dependent on these binding affinities and the formation of bond type between ligand and receptor complexes (Rozas, 2007). The metabolites with binding affinities near the dimer’s DNA binding sites (Supplementary Figure S1) indicate that the compounds might have anti-inflammatory properties by preventing activated NF-ĸβ (p65) from binding to DNA (Chen et al., 1998). Thus, molecular docking results indicate that bonding interactions and their characterization are a step toward new NF-ĸβ (p65) blocker development in a medicinal approach to respiratory problems. However, further intensive studies are required to authenticate the inhibition of transcription factors.

## 5 Conclusion

GC-MS analysis expressed the presence of 24 different secondary constituents that belong to different classes, such as anti-oxidants and anti-inflammatories. *In vivo* and *in vitro* experimental outcomes showed a marked reduction in of neutrophil and macrophage infiltration, pulmonary edema, improved SO_2_ levels, and restoration of anti-oxidant and anti-inflammatory markers. The AChE inhibitory profile indicates the use of *Pb.Cr* to treat inflammatory conditions. These findings offer a solid scientific basis for the anti-oxidant as well as anti-inflammatory properties of *Pb.Cr* by primarily inhibiting NF-ĸβ (p65) and shielding the lungs. Our research provides future insights into the potential of *P. bistorta* root for pharmacological breakthroughs to treat ALI/ARDS.

## Data Availability

The datasets presented in this study can be found in online repositories. The names of the repository/repositories and accession number(s) can be found in the article/Supplementary Material.

## Abbreviations

ABTS, 2,2′-azino-bis(3-ethylbenzothiazoline-6-sulfonic acid); AChE, acetylcholinesterase; ADME, absorption, distribution, metabolism and excretion; ANOVA, one way analysis of variance; cDNA, complimentary DNA; ALI, acute lung injury; AlCl_3,_ aluminum chloride; ARDS, acute respiratory distress syndrome; BALF, bronchoalveolar lavage fluid; BChE, butyrylcholinesterase; CNS, central nervous system; COX-2, cyclo-oxygenase-2; ℃, degrees Celsius; dH_2_O, distilled water; DAD, diffuse alveolar damage; Dexa, dexamethasone; DLC, differential leukocyte count; DPPH, 2,2-diphenyl-1-picrylhydrazyl; DTNB, 5,5′-dithiobis-(2-nitrobenzoic acid; EACML, elevated alveolar-capillary membrane leakage; eV, electron volts; FC, Folin–Ciocalteu; GAE, gallic acid equivalent; GC-MS, gas chromatography mass spectrometry; Hrs, hours; H & E, hematoxylin and eosin; IL-1β, interleukin-1 beta; IL-6, interleukin-6; IC_50_, half maximal inhibitory concentration; ICU, intensive care unit; i.t., intra-tracheal; IUB, Islamia University of Bahawalpur; KH_2_PO_4_, potassium dihydrogen phosphate; LD_50_, median lethal dose; LPS, lipo-polysaccharide; µL, microliter; mL, milliliter; min., minute; mg/kg, milligram per kilogram; NaOH, sodium hydroxide; NaNO_2_, sodium nitrite; NF-ĸβ, nuclear factor kappa beta; NIST, National Institute of Standards and Technology; Nm, nanometer; NS, normal saline; PAEC, Pharmacy Animal Ethics Committee; *Pb.Cr,* crude hydro-methanolic extract of *P. bistorta* L.; PBS, phosphate buffer solution; PDB, Protein Data Bank; PEEP, positive end-expiratory pressure; P.O., per oral; QE, quercetin equivalent; SEM, standard error of mean; SD, standard deviation; SO_2_, oxygen saturation; SOD, super oxide dismutase; TAC, total anti-oxidant capacity; TFC, total flavonoid contents; TNF-α, tumor necrosis factor-alpha; TOS, total oxidative status; TPC, total phenolic contents; qRT-PCR, quantitative reverse transcriptase polymerase chain reaction; %, percent; 2D, two dimensional; 3D, three dimensional.
