# Supplementary material for: Exploration of the anti-inflammatory potential of Polygonum bistorta L.: protection against LPS-induced acute lung injury in rats via NF-ĸβ pathway inhibition
Source: Front Pharmacol. 2025 Feb 5;15:1500085. doi: 10.3389/fphar.2024.1500085 (PMC11851016; doi:10.3389/fphar.2024.1500085)
Supplement: Supplementary file 1 [file DataSheet2.pdf]

Library Search Report

```
Data Path :MUBASHIR AZIZ\Data File
Acq On    : 23 Oct 2023  13:37
Sample    : Pb
Cr
Misc      :
ALS Vial  : 2   Sample Multiplier: 1

Search Libraries:   C:\MassHunter\LIBRARY\NIST20.L     Minimum Quality:   0

Unknown Spectrum:   Apex
Integration Events: ChemStation Integrator - autoint1.e
```

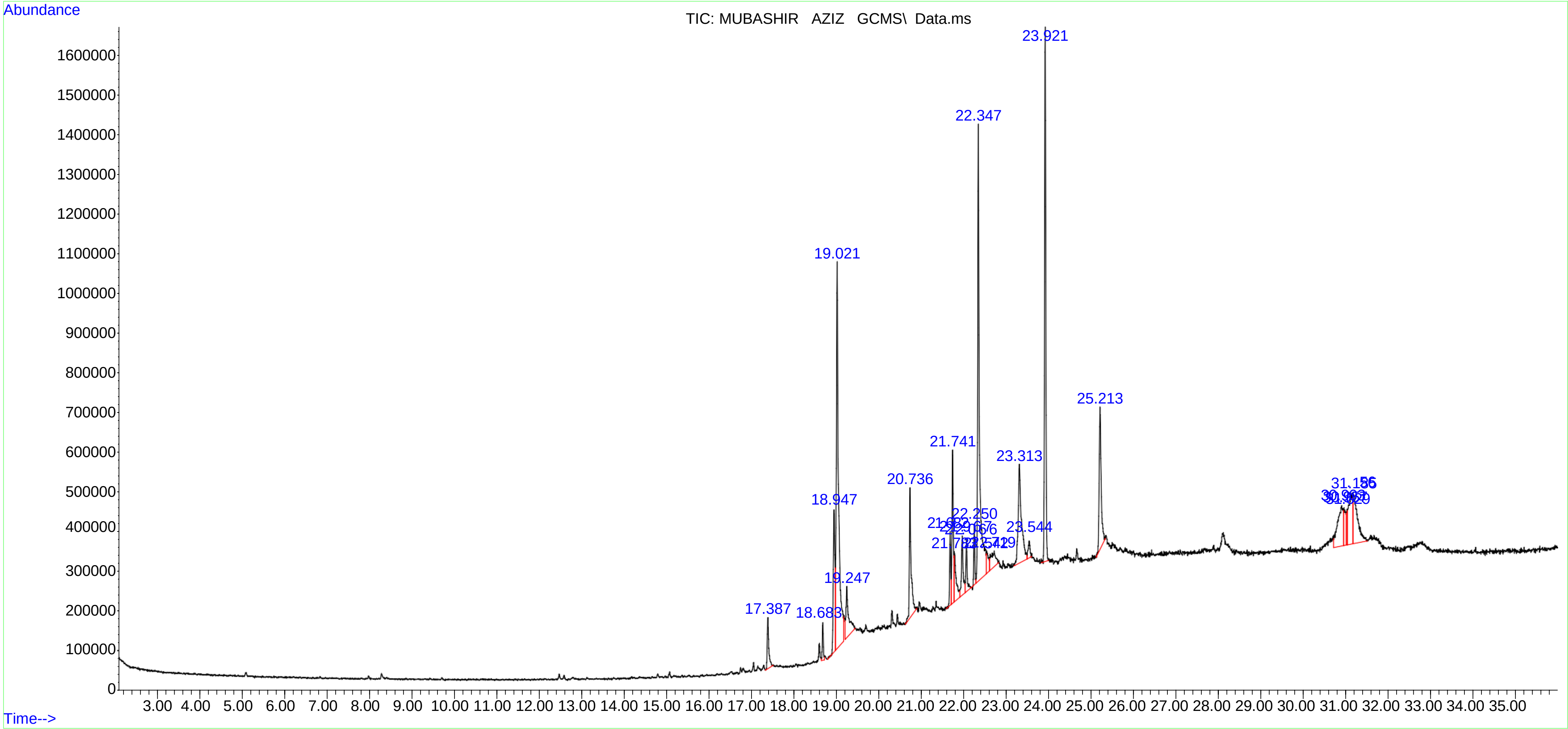

Unknown Spectrum based on Apex

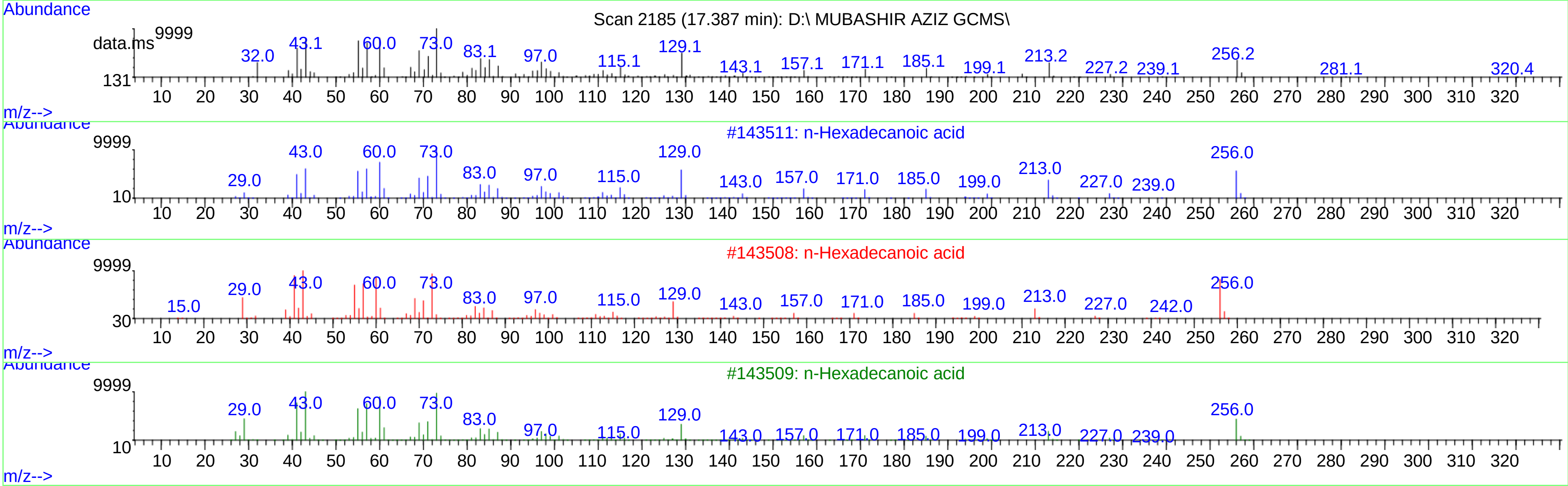

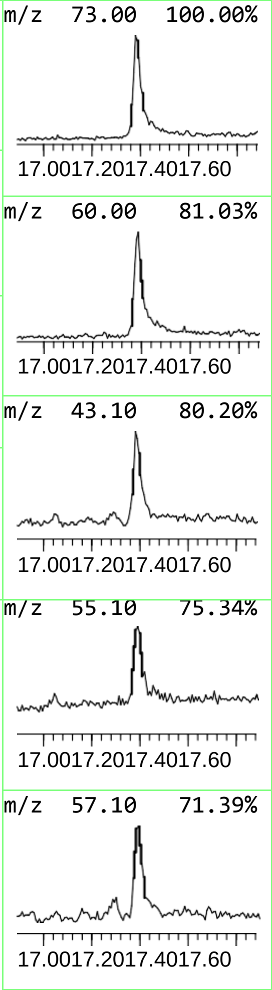

Data File: D:\ MUBASHIR AZIZ GCMS\ DATA
Sample : Pb Cr

Peak Number: 1 at 17.387 min Area: 3339363 Area % 1.54

The 3 best hits from each library.

C:\MassHunter\LIBRARY\NIST20.L

	The 3 best hits from each library.	Ref\#	CAS\#	Qual
1	n-Hexadecanoic acid	143511	000057-10-3	99
2	n-Hexadecanoic acid	143508	000057-10-3	99
3	n-Hexadecanoic acid	143509	000057-10-3	95

Unknown Spectrum based on Apex

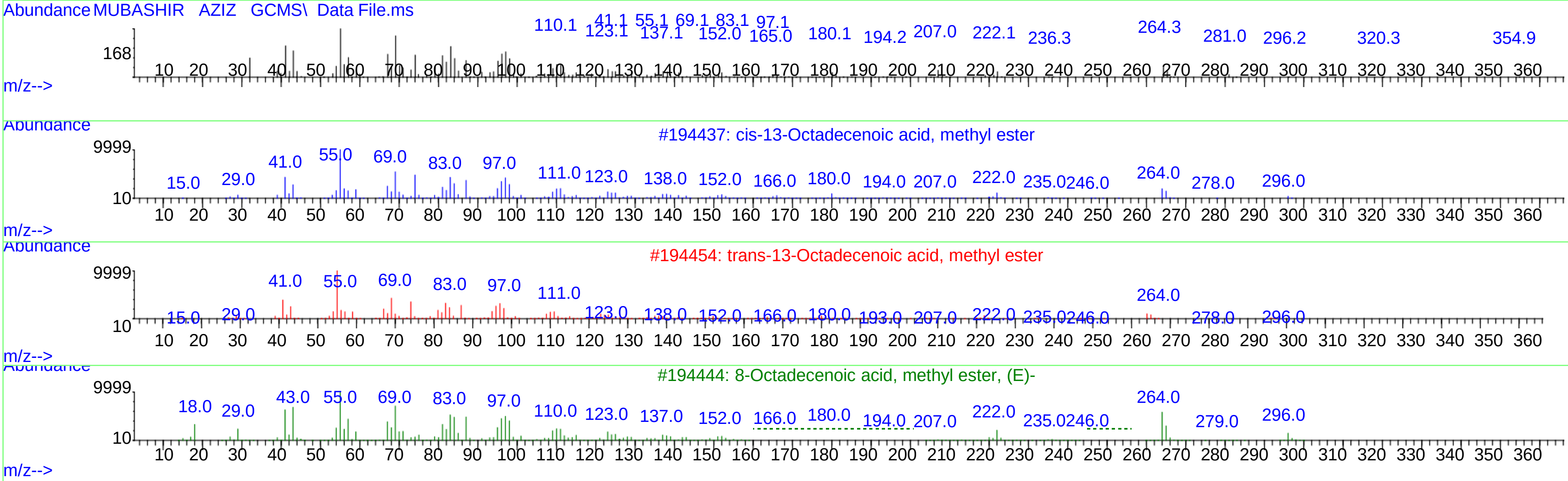

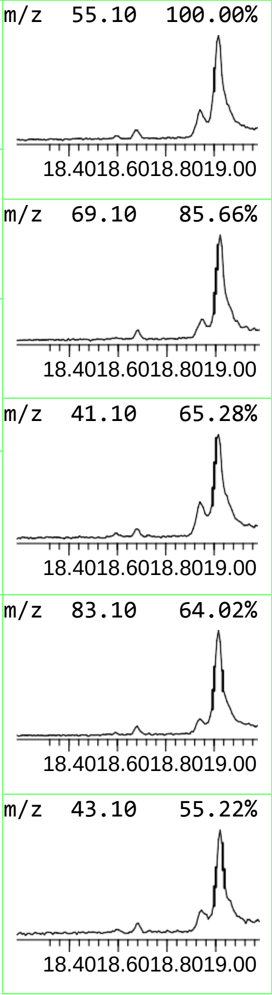

Data File: D:\ MUBASHIR AZIZ GCMS\ DATA
Sample : Pb Cr

Peak Number: 2 at 18.683 min Area: 1638315 Area % 0.76

The 3 best hits from each library. Ref\# CAS\# Qual

C:\MassHunter\LIBRARY\NIST20.L

	Compound	Ref#	CAS#	Qual
1	cis-13-Octadecenoic acid, methyl...	194437	1010333-58-3	99
2	trans-13-Octadecenoic acid, meth...	194454	1000333-61-3	97
3	8-Octadecenoic acid, methyl este...	194444	026528-50-7	96

Unknown Spectrum based on Apex

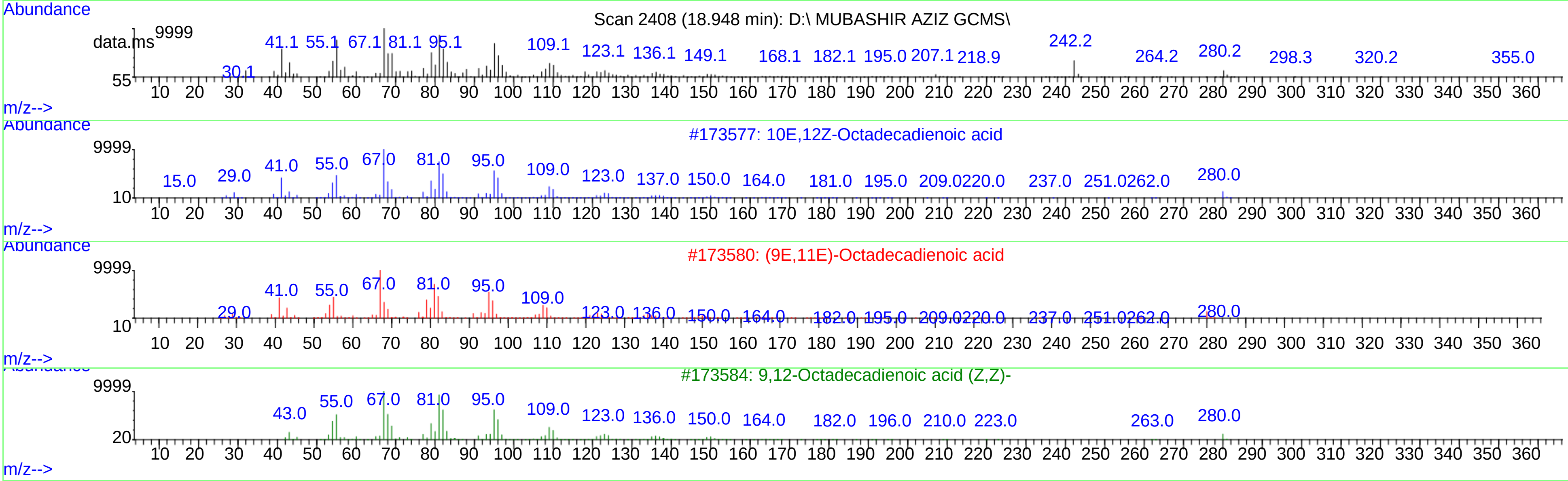

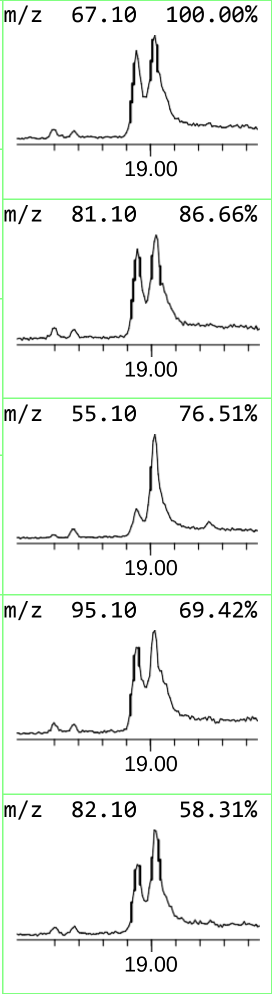

Data File: D:\ MUBASHIR AZIZ GCMS\ DATA
Sample : Pb Cr

Peak Number: 3 at 18.947 min Area: 9304764 Area % 4.30

The 3 best hits from each library.

C:\MassHunter\LIBRARY\NIST20.L

	Name	Ref\#	CAS\#	Qual
1	10E,12Z-Octadecadienoic acid	173577	002420-56-6	99
2	(9E,11E)-Octadecadienoic acid	173580	000544-71-8	99
3	9,12-Octadecadienoic acid (Z,Z)-	173584	000060-33-3	99

Unknown Spectrum based on Apex

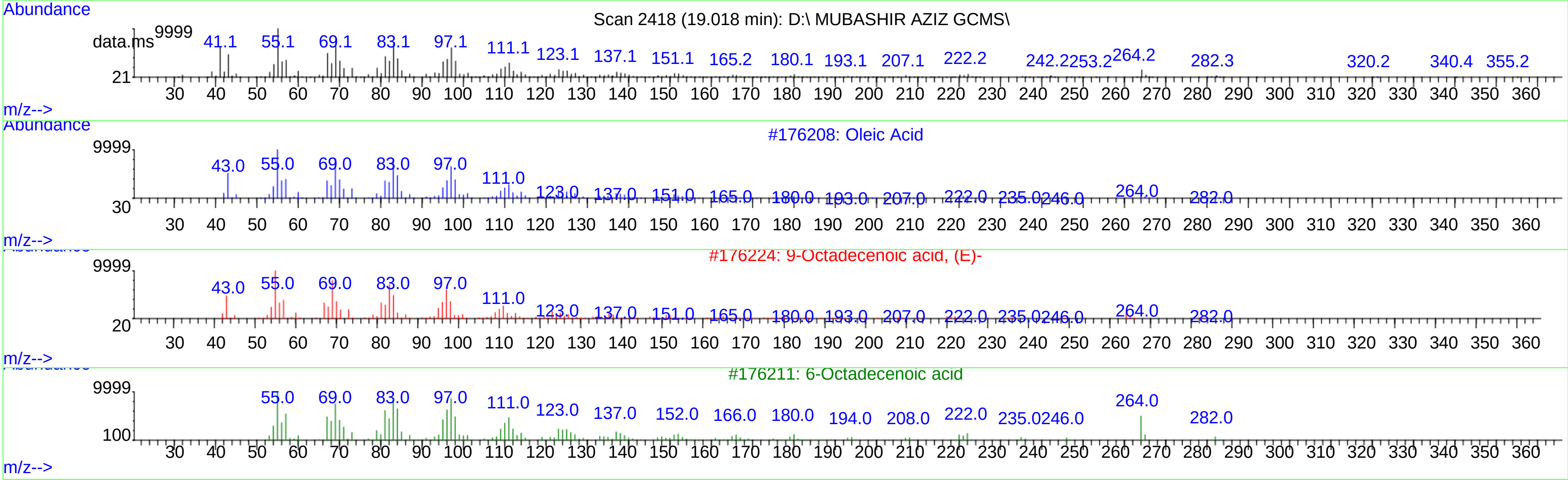

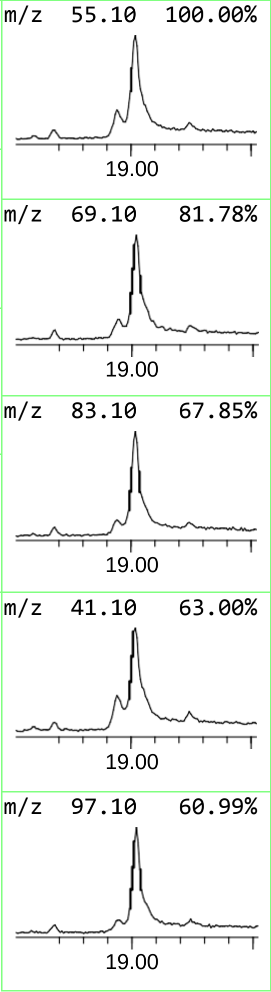

Data File: D:\ MUBASHIR AZIZ GCMS\ DATA
Sample : Pb Cr

Peak Number: 4 at 19.021 min Area: 36274361 Area % 16.77

The 3 best hits from each library. Ref# CAS# Qual

C:\MassHunter\LIBRARY\NIST20.L

		Ref#	CAS#	Qual
1	Oleic Acid	176208	000112-80-1	99
2	9-Octadecenoic acid, (E)-	176224	000112-79-8	99
3	6-Octadecenoic acid	176211	1000336-66-8	99

Unknown Spectrum based on Apex

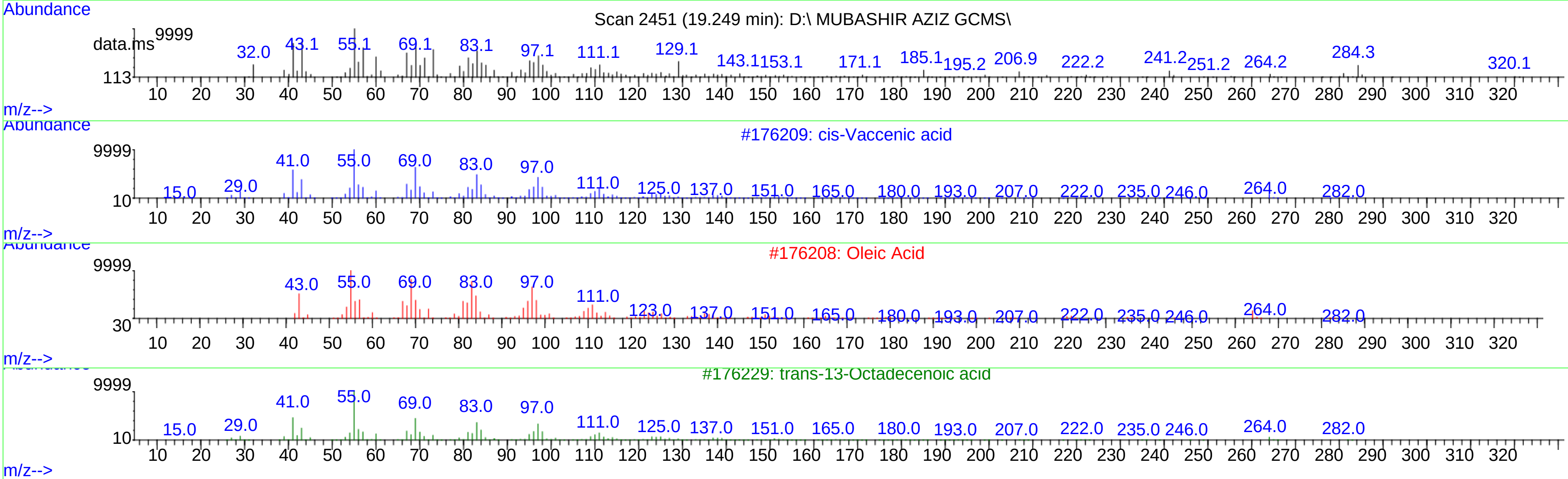

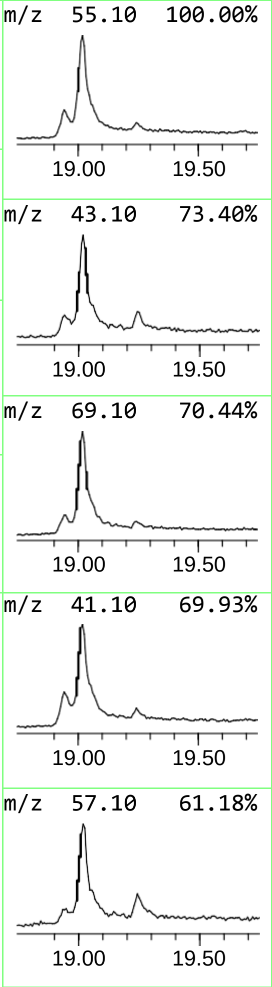

Data File: D:\ MUBASHIR AZIZ GCMS\ DATA
Sample : Pb Cr

Peak Number: 5 at 19.247 min Area: 5808823 Area % 2.69

The 3 best hits from each library.	Ref#	CAS#	Qual
C:\MassHunter\LIBRARY\NIST20.L			
1 cis-Vaccenic acid	176209	000506-17-2	93
2 Oleic Acid	176208	000112-80-1	86
3 trans-13-Octadecenoic acid	176229	000693-71-0	86

Unknown Spectrum based on Apex

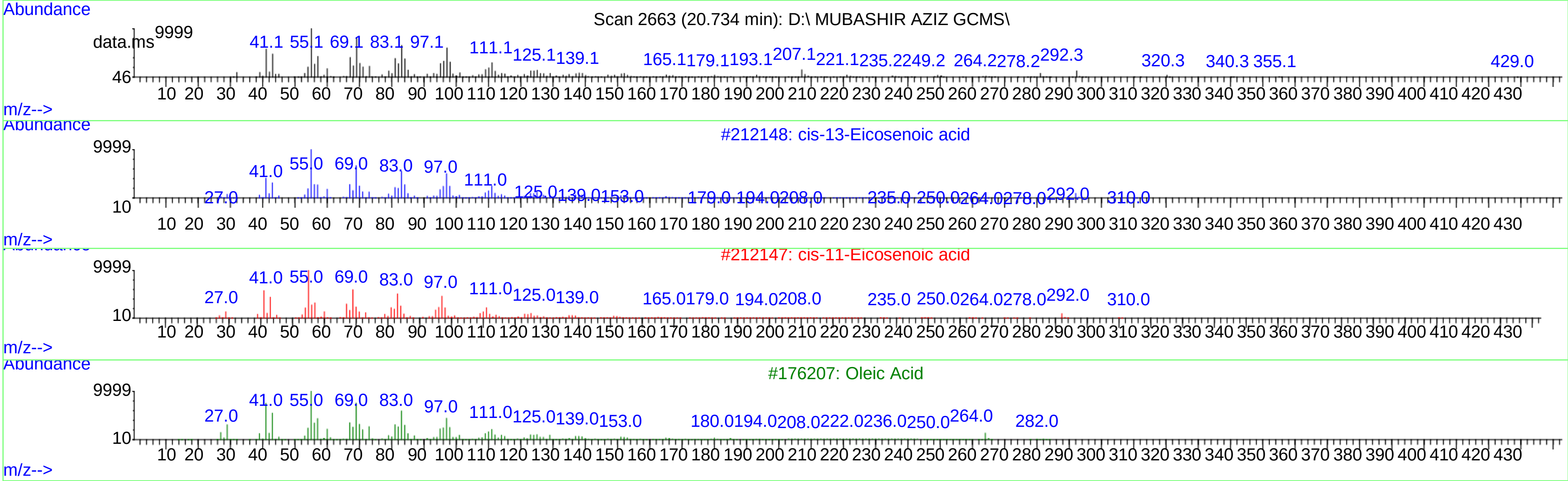

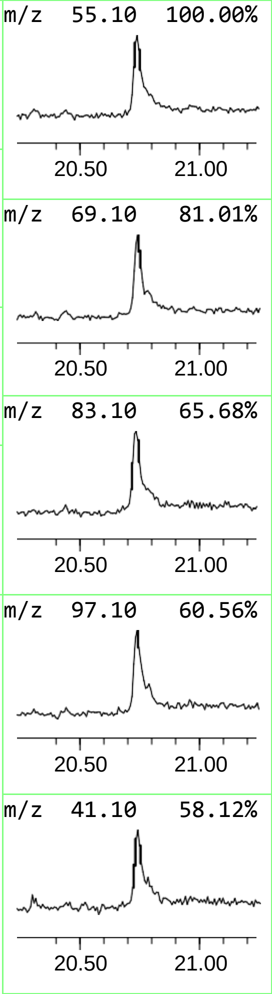

Data File: D:\ MUBASHIR AZIZ GCMS\ DATA
Sample : Pb Cr

Peak Number: 6 at 20.736 min Area: 9347128 Area % 4.32

The 3 best hits from each library.

		Ref\#	CAS\#	Qual
C:\MassHunter\LIBRARY\NIST20.L				
1	cis-13-Eicosenoic acid	212148	017735-94-3	99
2	cis-11-Eicosenoic acid	212147	005561-99-9	99
3	Oleic Acid	176207	000112-80-1	93

Unknown Spectrum based on Apex

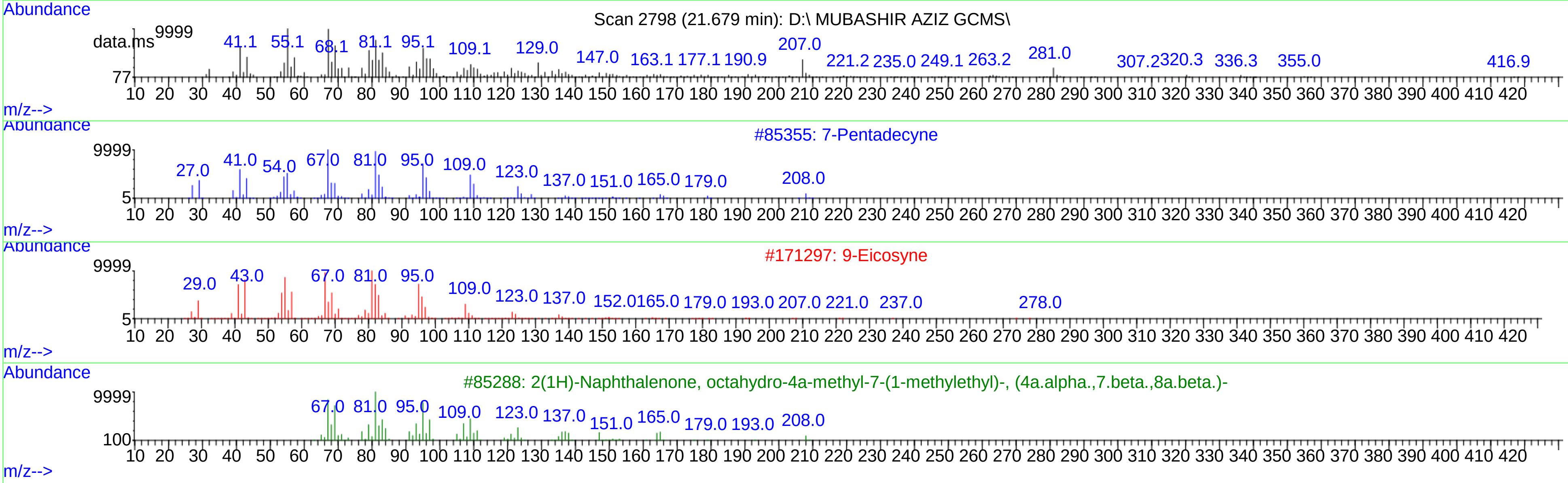

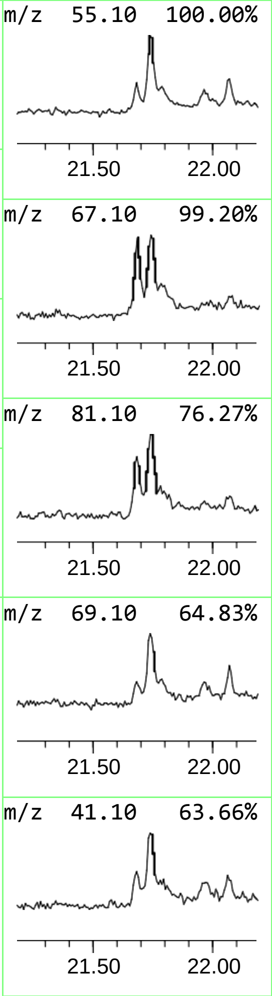

Data File: D:\ MUBASHIR AZIZ GCMS\ DATA
Sample : Pb Cr

Peak Number: 7 at 21.682 min Area: 3338923 Area % 1.54

The 3 best hits from each library.	Ref\#	CAS\#	Qual
C:\MassHunter\LIBRARY\NIST20.L			
1 7-Pentadecyne	85355	022089-89-0	87
2 9-Eicosyne	171297	071899-38-2	86
3 2(1H)-Naphthalenone, octahydro-4...	85288	054594-42-2	83

Unknown Spectrum based on Apex

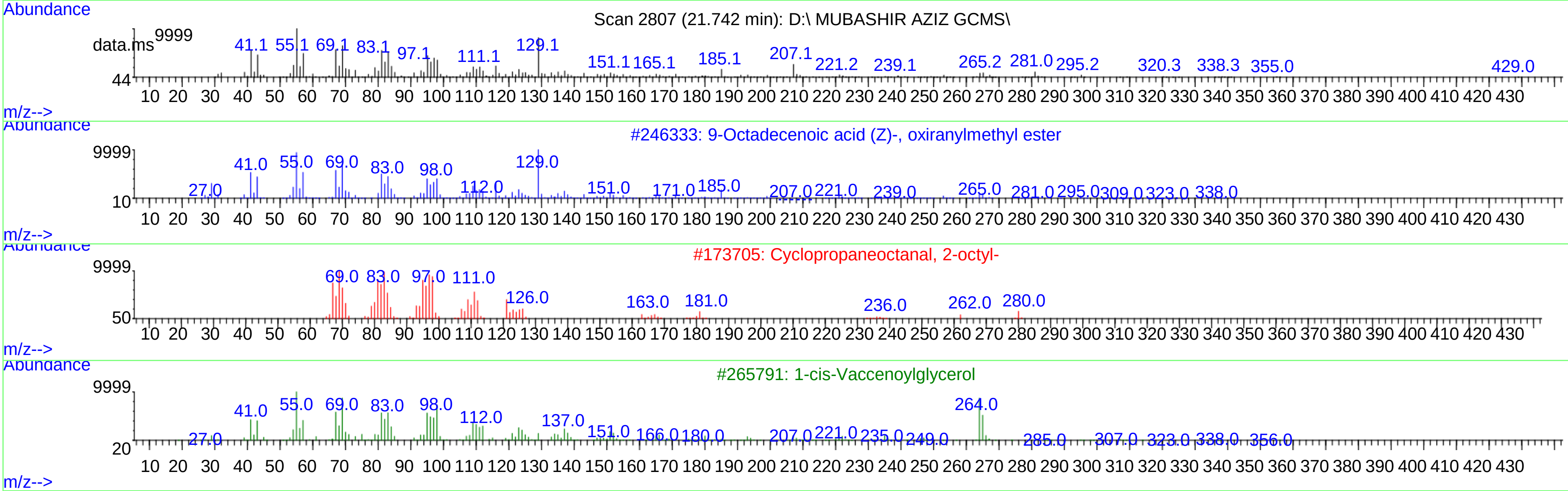

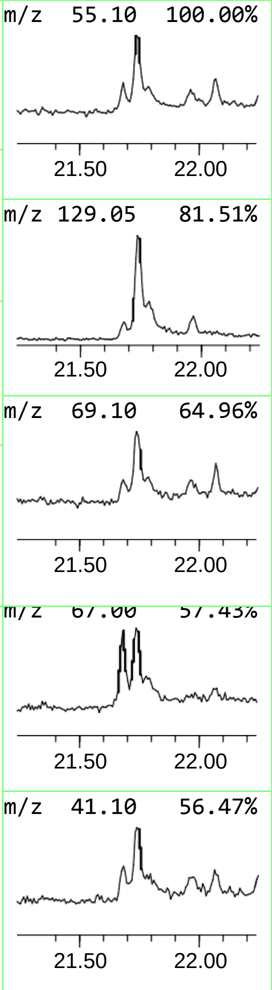

Data File: D:\ MUBASHIR AZIZ GCMS\ DATA
Sample : Pb Cr

Peak Number: 8 at 21.741 min Area: 8495451 Area % 3.93

The 3 best hits from each library.

C:\MassHunter\LIBRARY\NIST20.L

		Ref\#	CAS\#	Qual
1	9-Octadecenoic acid (Z)-, oxiran...	246333	005431-33-4	99
2	Cyclopropaneoctanal, 2-octyl-	173705	056196-06-6	94
3	1-cis-Vaccenoylglycerol	265791	020379-67-3	92

Unknown Spectrum based on Apex

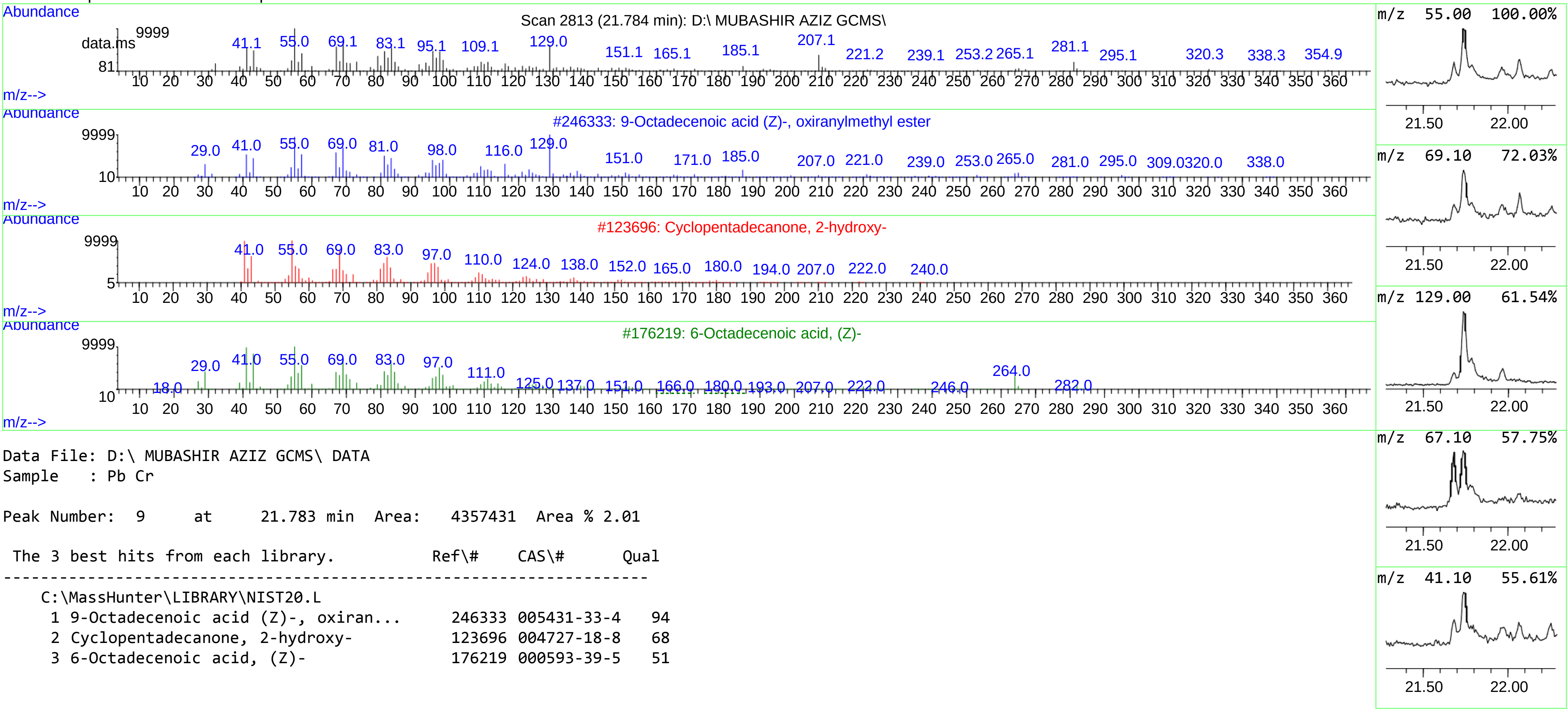

Data File: D:\ MUBASHIR AZIZ GCMS\ DATA
Sample : Pb Cr

Peak Number: 9 at 21.783 min Area: 4357431 Area % 2.01

The 3 best hits from each library.

C:\MassHunter\LIBRARY\NIST20.L

		Ref\#	CAS\#	Qual
1	9-Octadecenoic acid (Z)-, oxiran...	246333	005431-33-4	94
2	Cyclopentadecanone, 2-hydroxy-	123696	004727-18-8	68
3	6-Octadecenoic acid, (Z)-	176219	000593-39-5	51

Unknown Spectrum based on Apex

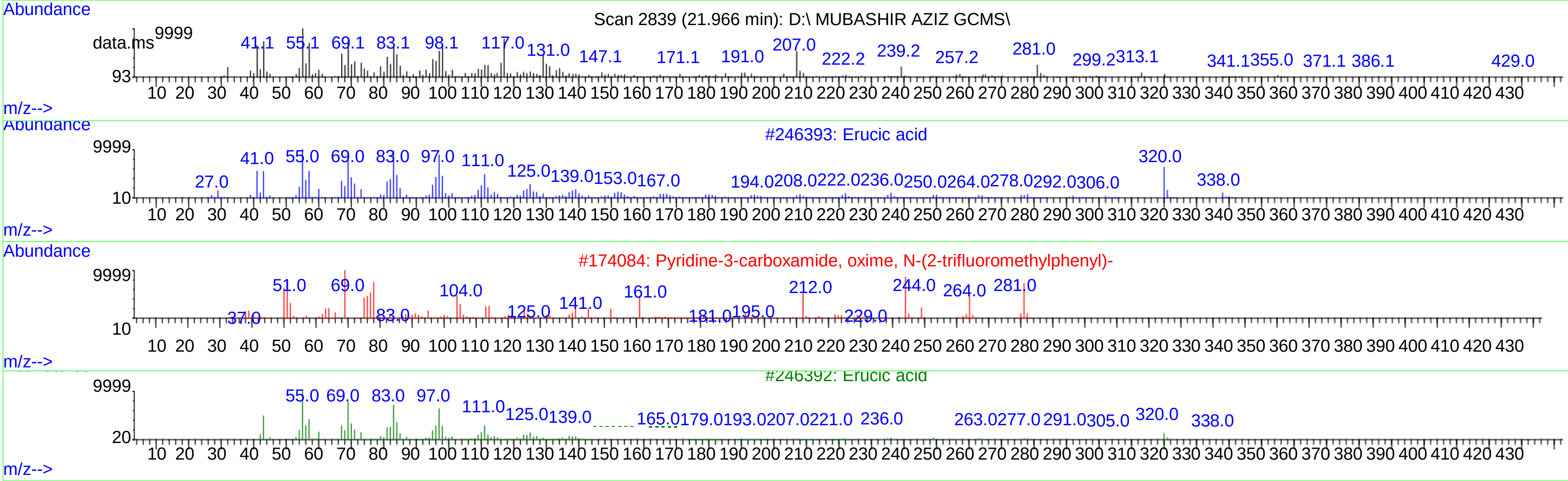

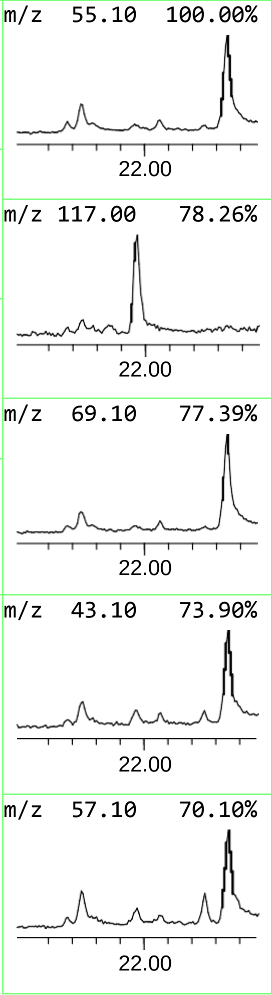

Data File: D:\ MUBASHIR AZIZ GCMS\ DATA
Sample : Pb Cr

Peak Number: 10 at 21.967 min Area: 4155790 Area % 1.92

The 3 best hits from each library.

C:\MassHunter\LIBRARY\NIST20.L

	The 3 best hits from each library.	Ref\#	CAS\#	Qual
1	Erucic acid	246393	000112-86-7	83
2	Pyridine-3-carboxamide, oxime, N...	174084	288246-53-7	59
3	Erucic acid	246392	000112-86-7	55

Unknown Spectrum based on Apex

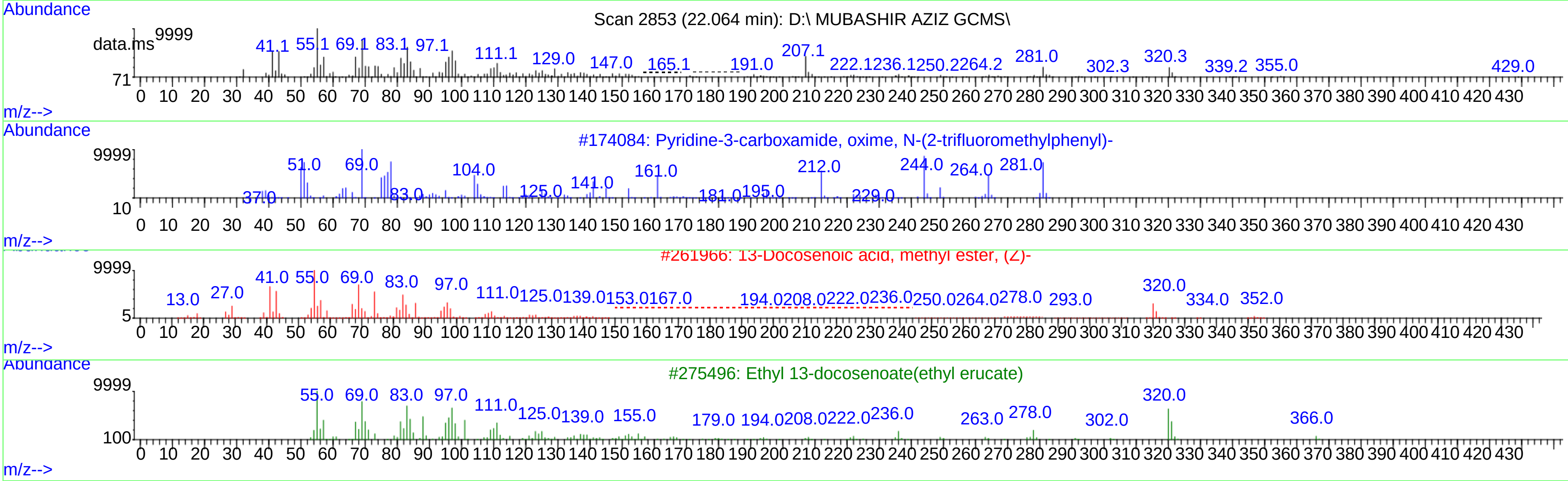

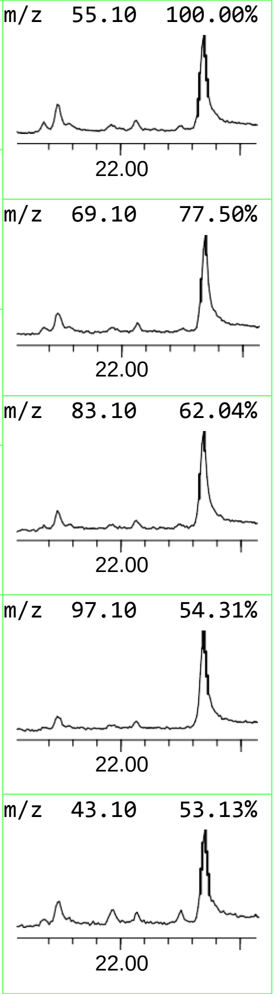


```
Data File: D:\ MUBASHIR AZIZ GCMS\ DATA
Sample    : Pb Cr

Peak Number:  11    at     22.066 min   Area:    2836981   Area % 1.31

 The 3 best hits from each library.          Ref\#    CAS\#      Qual
-----------------------------------------------------------------
    C:\MassHunter\LIBRARY\NIST20.L
     1 Pyridine-3-carboxamide, oxime, N...      174084 288246-53-7   91
     2 13-Docosenoic acid, methyl ester...      261966 001120-34-9   86
     3 Ethyl 13-docosenoate(ethyl erucate)      275496 037910-77-3   68
```

Unknown Spectrum based on Apex

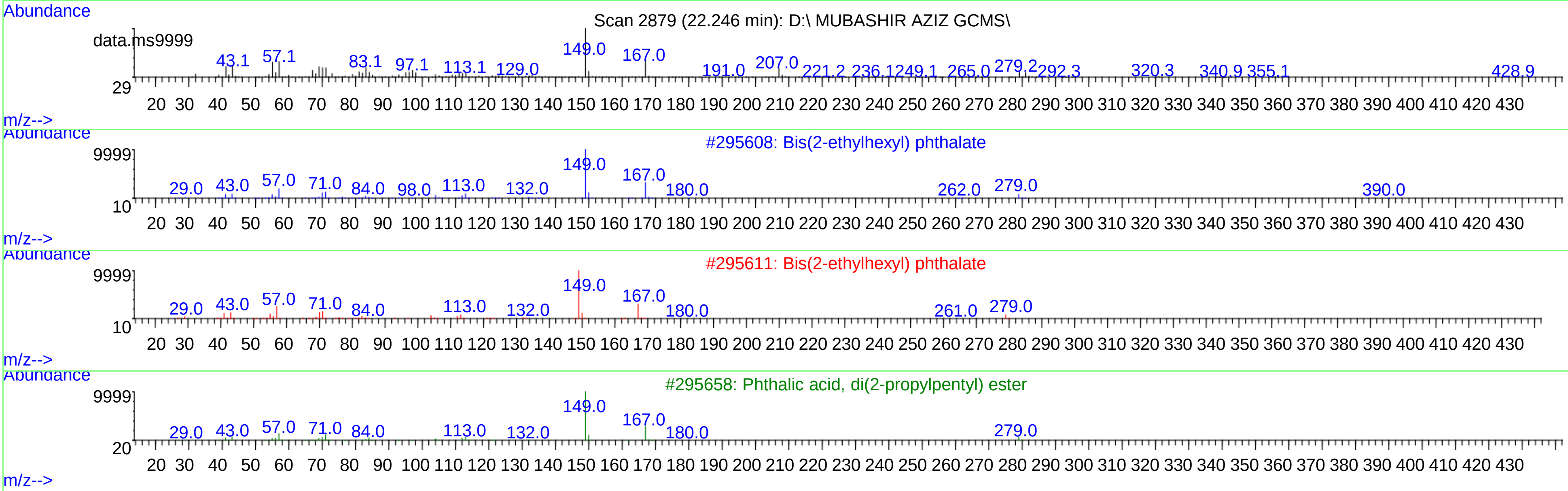

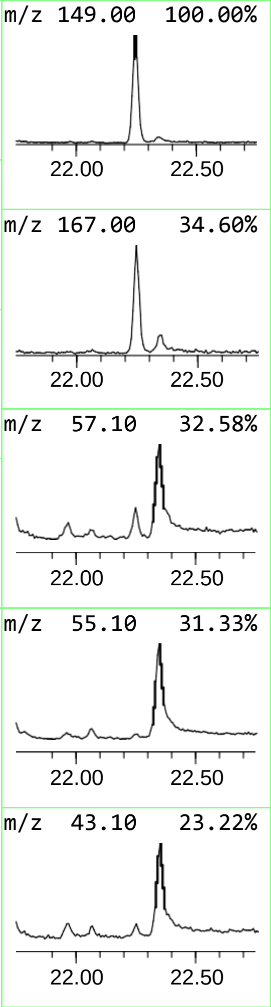

Data File: D:\ MUBASHIR AZIZ GCMS\ DATA
Sample : Pb Cr

Peak Number: 12 at 22.250 min Area: 2319123 Area % 1.07

The 3 best hits from each library.	Ref\#	CAS\#	Qual
C:\MassHunter\LIBRARY\NIST20.L			
1 Bis(2-ethylhexyl) phthalate	295608	000117-81-7	64
2 Bis(2-ethylhexyl) phthalate	295611	000117-81-7	64
3 Phthalic acid, di(2-propylpentyl...	295658	1000377-93-5	64

Unknown Spectrum based on Apex

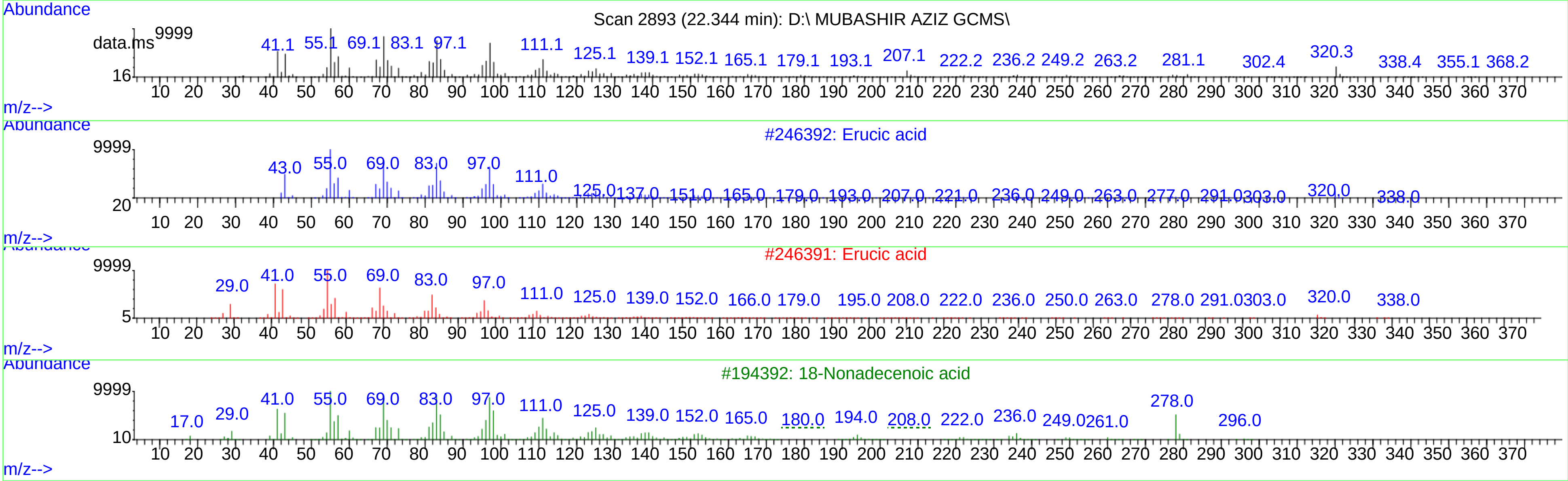

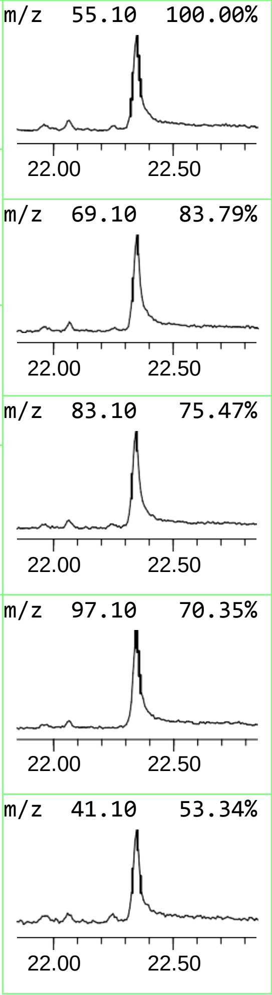

Data File: D:\ MUBASHIR AZIZ GCMS\ DATA
Sample : Pb Cr

Peak Number: 13 at 22.347 min Area: 33712736 Area % 15.58

The 3 best hits from each library.

C:\MassHunter\LIBRARY\NIST20.L

	Ref#	CAS#	Qual
1 Erucic acid	246392	000112-86-7	99
2 Erucic acid	246391	000112-86-7	96
3 18-Nonadecenoic acid	194392	076998-87-3	95

Unknown Spectrum based on Apex

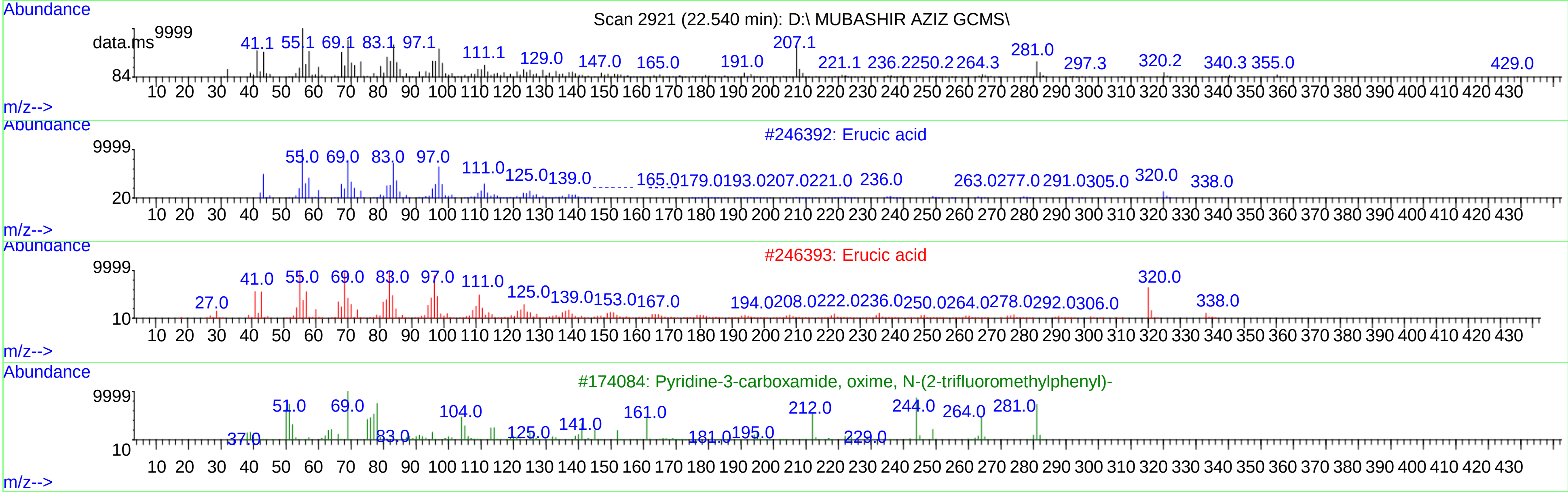

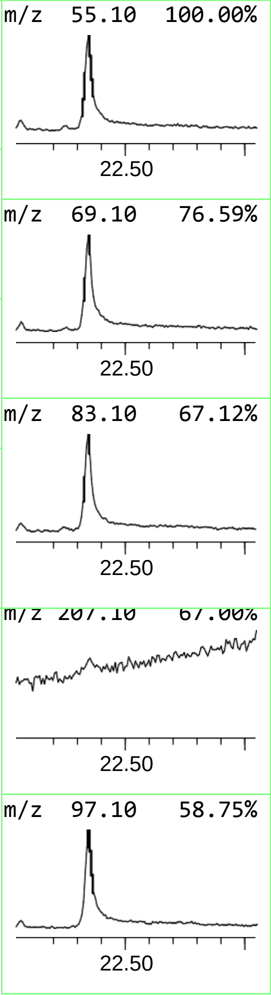

Data File: D:\ MUBASHIR AZIZ GCMS\ DATA
Sample : Pb Cr

Peak Number: 14 at 22.542 min Area: 1948438 Area % 0.90

The 3 best hits from each library.	Ref\#	CAS\#	Qual
C:\MassHunter\LIBRARY\NIST20.L			
1 Erucic acid	246392	000112-86-7	99
2 Erucic acid	246393	000112-86-7	99
3 Pyridine-3-carboxamide, oxime, N...	174084	288246-53-7	94

Unknown Spectrum based on Apex

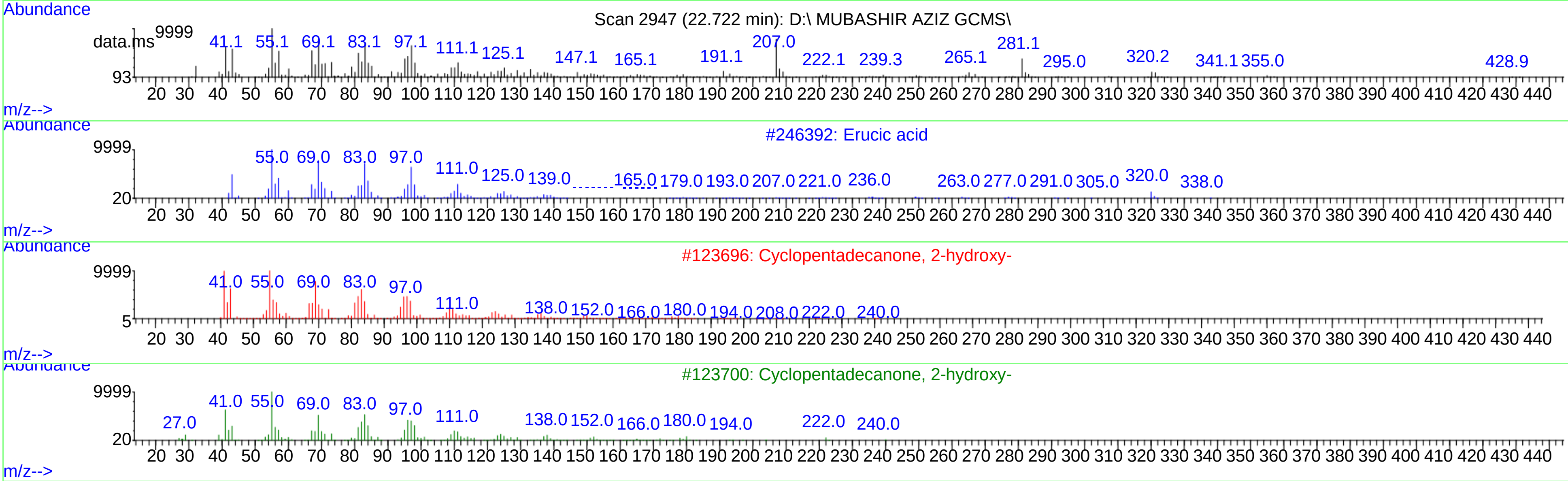

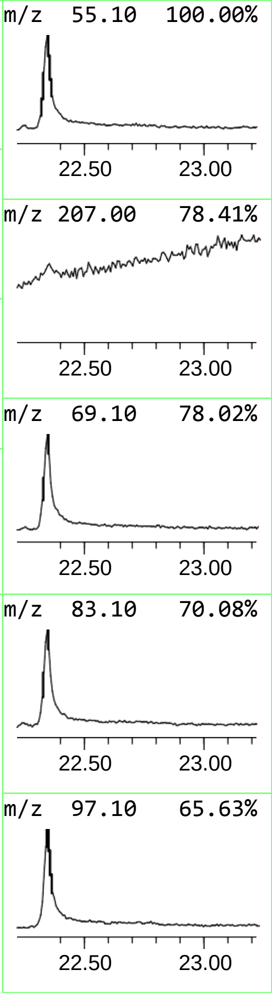

Data File: D:\ MUBASHIR AZIZ GCMS\ DATA
Sample : Pb Cr

Peak Number: 15 at 22.719 min Area: 3196084 Area % 1.48

The 3 best hits from each library.

C:\MassHunter\LIBRARY\NIST20.L

		Ref\#	CAS\#	Qual
1	Erucic acid	246392	000112-86-7	95
2	Cyclopentadecanone, 2-hydroxy-	123696	004727-18-8	93
3	Cyclopentadecanone, 2-hydroxy-	123700	004727-18-8	90

Unknown Spectrum based on Apex

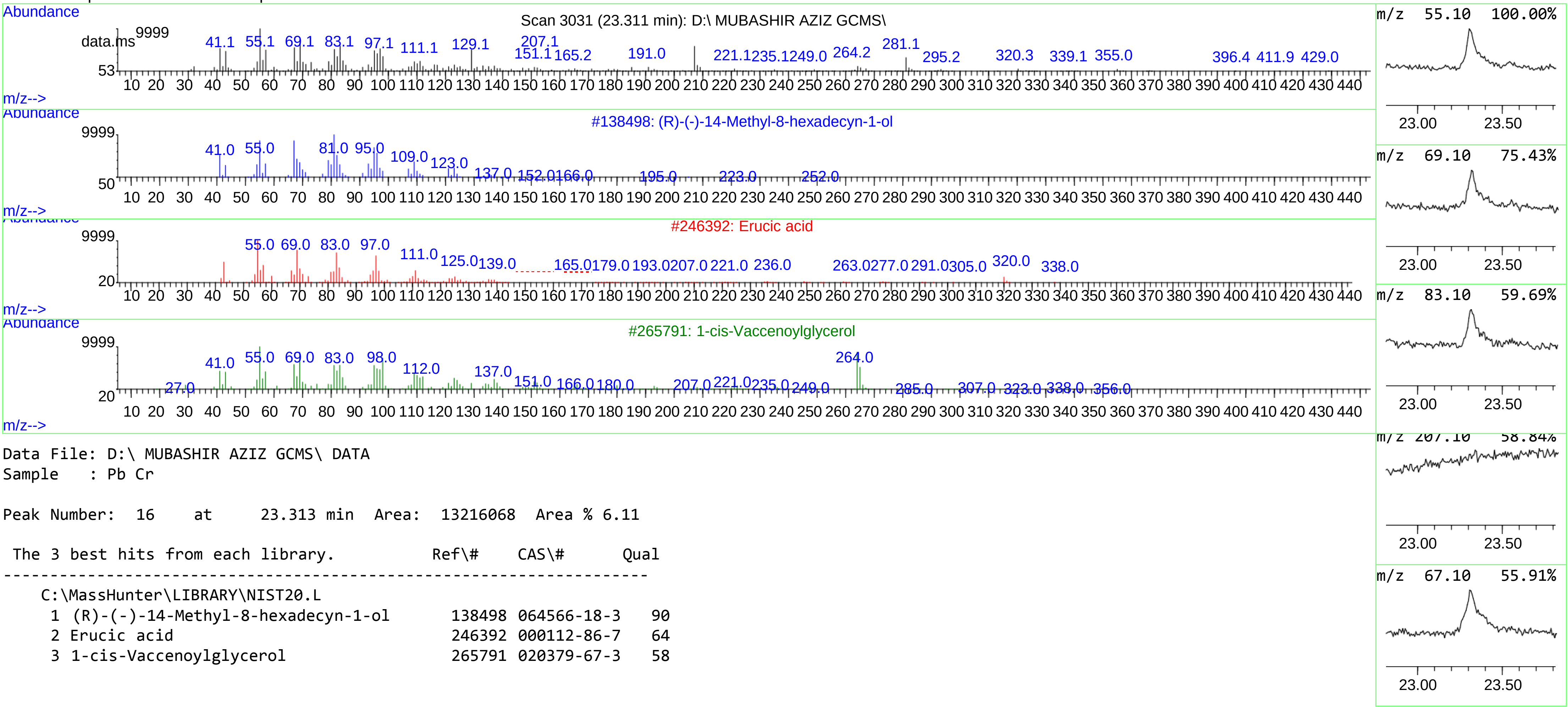

Data File: D:\ MUBASHIR AZIZ GCMS\ DATA
Sample : Pb Cr

Peak Number: 16 at 23.313 min Area: 13216068 Area % 6.11

The 3 best hits from each library.

C:\MassHunter\LIBRARY\NIST20.L

		Ref\#	CAS\#	Qual
1	(R)-(-)-14-Methyl-8-hexadecyn-1-ol	138498	064566-18-3	90
2	Erucic acid	246392	000112-86-7	64
3	1-cis-Vaccenoylglycerol	265791	020379-67-3	58

Unknown Spectrum based on Apex

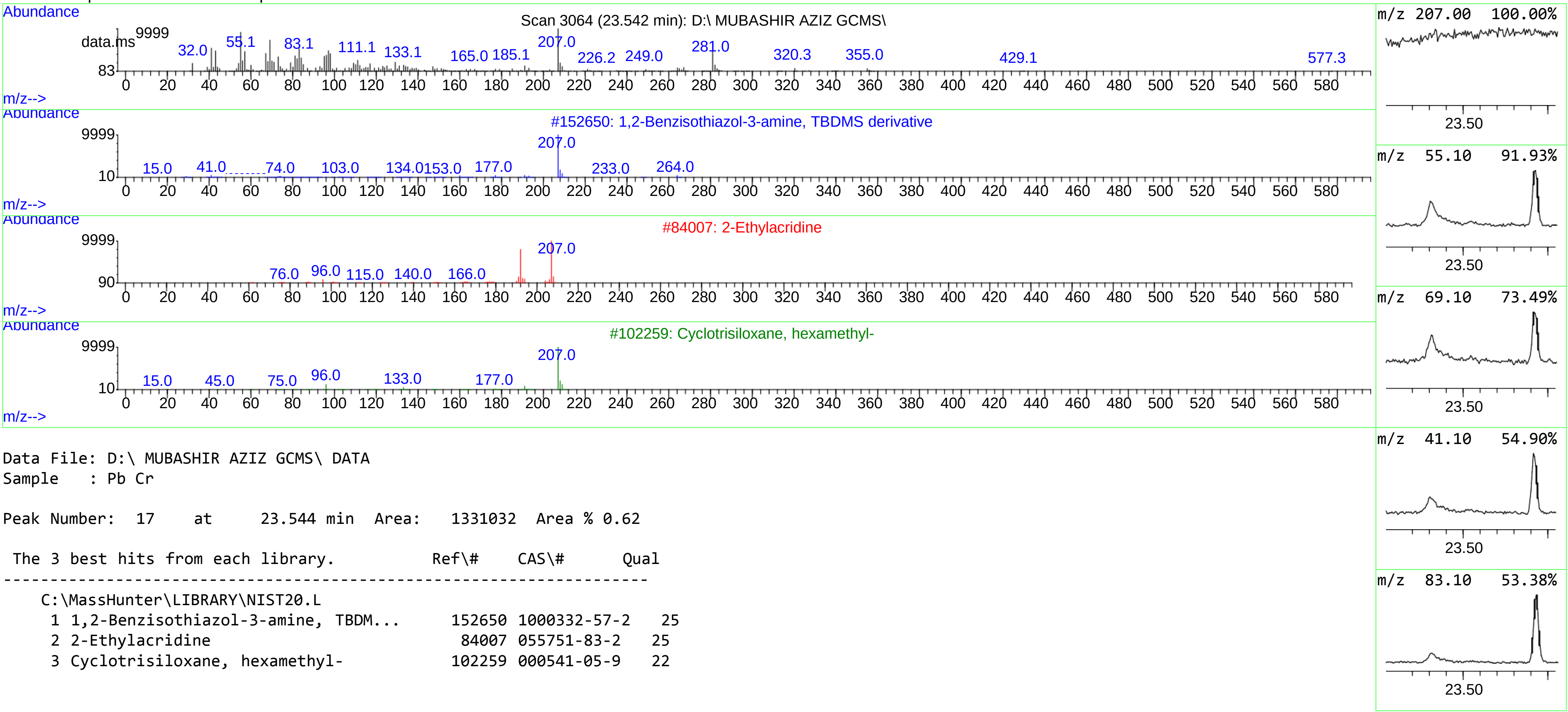

Data File: D:\ MUBASHIR AZIZ GCMS\ DATA
Sample : Pb Cr

Peak Number: 17 at 23.544 min Area: 1331032 Area % 0.62

The 3 best hits from each library.

C:\MassHunter\LIBRARY\NIST20.L

		Ref\#	CAS\#	Qual
1	1,2-Benzisothiazol-3-amine, TBDM...	152650	1000332-57-2	25
2	2-Ethylacridine	84007	055751-83-2	25
3	Cyclotrisiloxane, hexamethyl-	102259	000541-05-9	22

Unknown Spectrum based on Apex

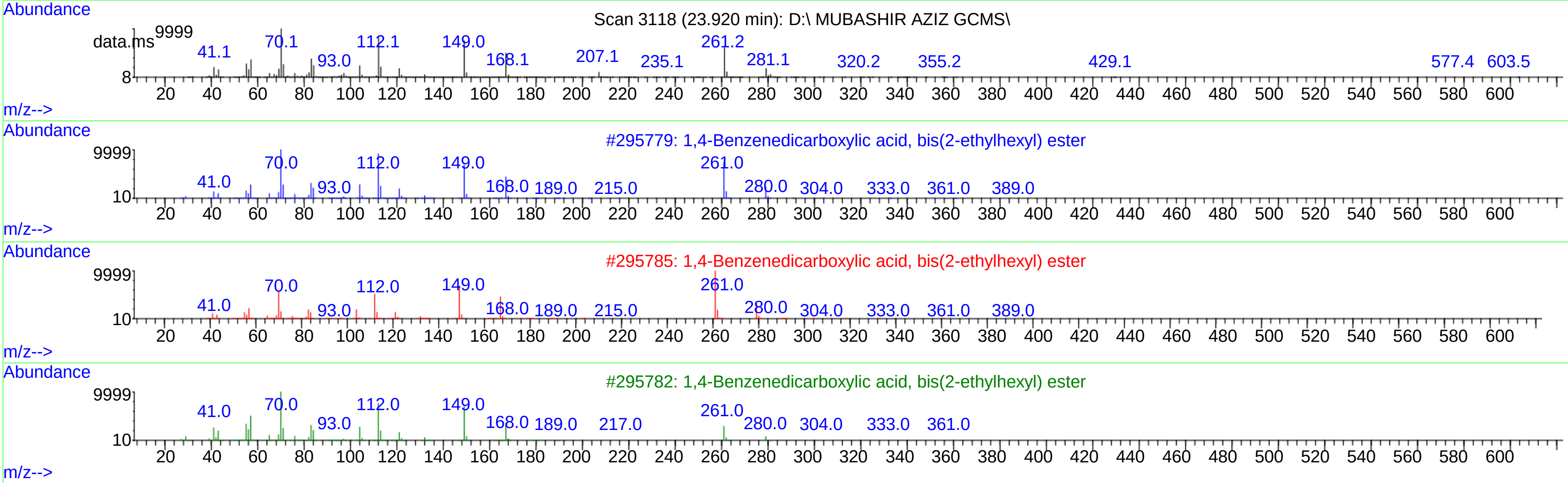

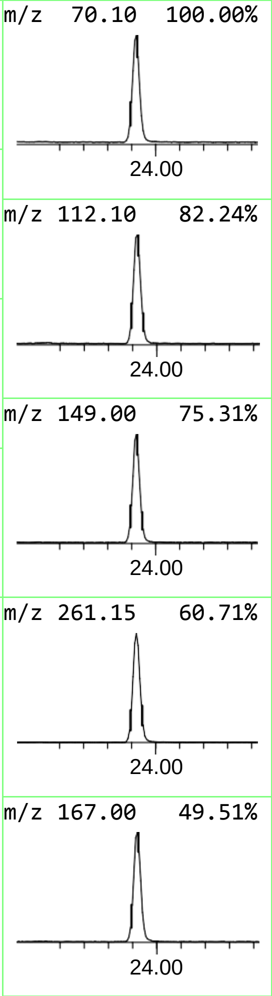

Data File: D:\ MUBASHIR AZIZ GCMS\ DATA
Sample : Pb Cr

Peak Number: 18 at 23.921 min Area: 28304857 Area % 13.08

The 3 best hits from each library.	Ref#	CAS#	Qual
C:\MassHunter\LIBRARY\NIST20.L			
1 1,4-Benzenedicarboxylic acid, bi...	295779	006422-86-2	95
2 1,4-Benzenedicarboxylic acid, bi...	295785	006422-86-2	91
3 1,4-Benzenedicarboxylic acid, bi...	295782	006422-86-2	91

Unknown Spectrum based on Apex

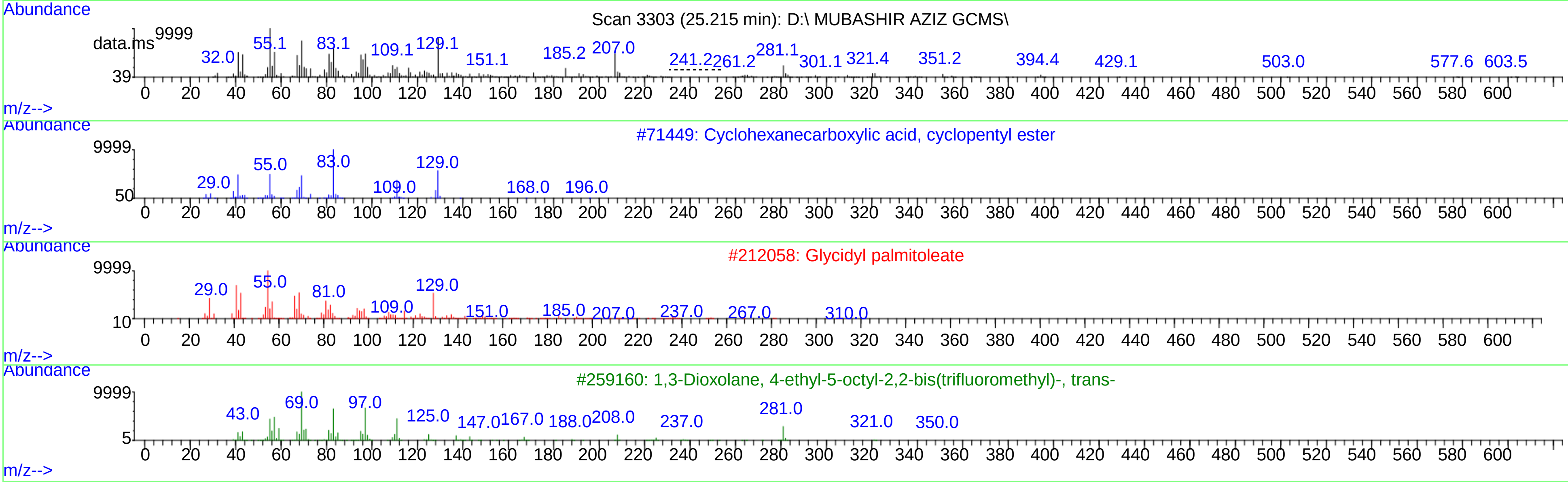

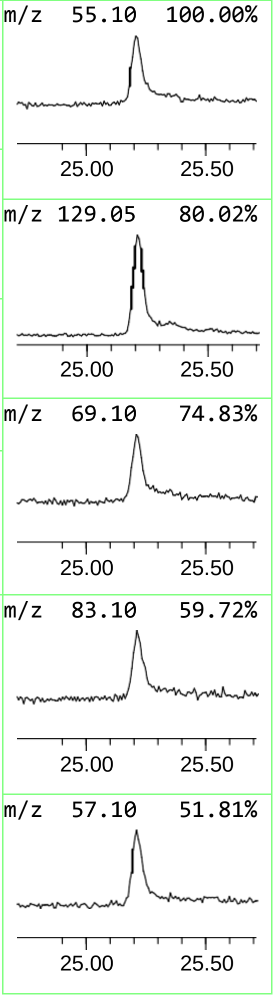

Data File: D:\ MUBASHIR AZIZ GCMS\ DATA
Sample : Pb Cr

Peak Number: 19 at 25.213 min Area: 11652305 Area % 5.39

The 3 best hits from each library.	Ref#	CAS#	Qual
C:\MassHunter\LIBRARY\NIST20.L			
1 Cyclohexanecarboxylic acid, cycl...	71449	005420-91-7	46
2 Glycidyl palmitoleate	212058	213738-77-3	44
3 1,3-Dioxolane, 4-ethyl-5-octyl-2...	259160	038274-73-6	35

Unknown Spectrum based on Apex

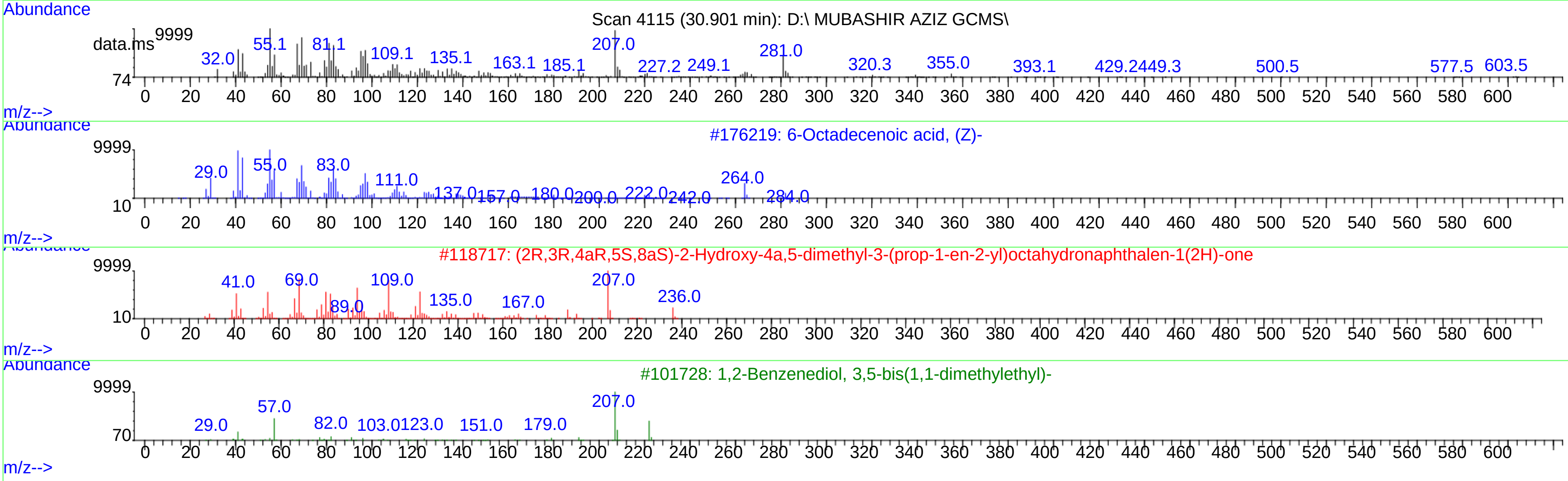

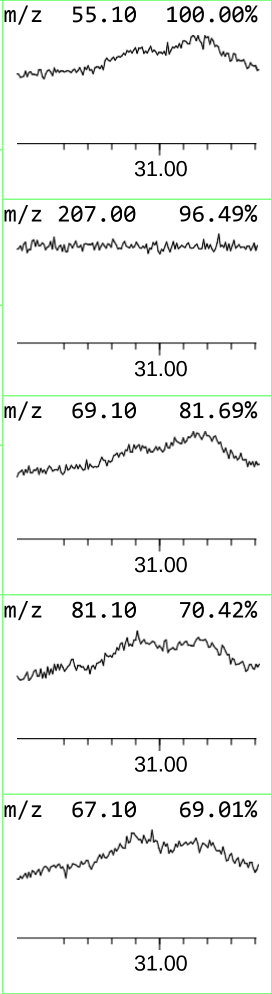

Data File: D:\ MUBASHIR AZIZ GCMS\ DATA
Sample : Pb Cr

Peak Number: 20 at 30.903 min Area: 9150411 Area % 4.23

The 3 best hits from each library.

C:\MassHunter\LIBRARY\NIST20.L

	Compound	Ref\#	CAS\#	Qual
1	6-Octadecenoic acid, (Z)-	176219	000593-39-5	53
2	(2R,3R,4aR,5S,8aS)-2-Hydroxy-4a,...	118717	066884-74-0	50
3	1,2-Benzenediol, 3,5-bis(1,1-dim...	101728	001020-31-1	43

Unknown Spectrum based on Apex

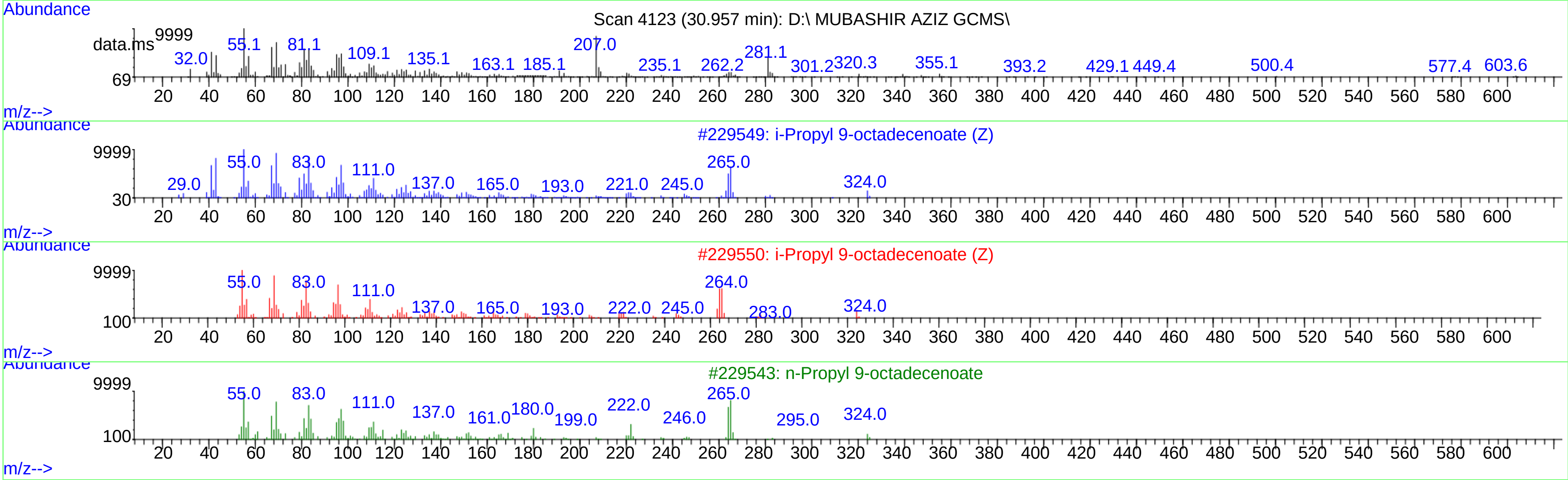

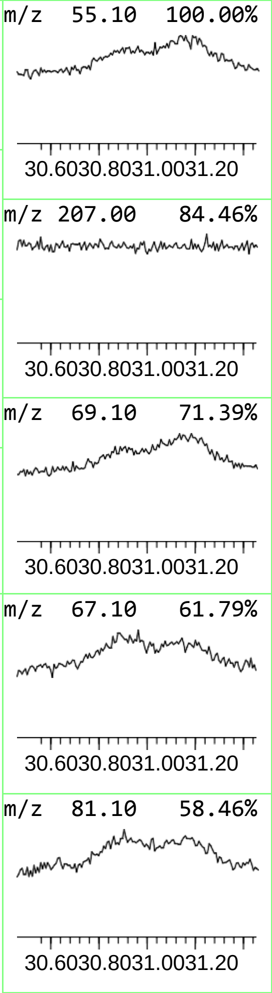

Data File: D:\ MUBASHIR AZIZ GCMS\ DATA
Sample : Pb Cr

Peak Number: 21 at 30.957 min Area: 3276165 Area % 1.51

The 3 best hits from each library.	Ref\#	CAS\#	Qual
C:\MassHunter\LIBRARY\NIST20.L			
1 i-Propyl 9-octadecenoate (Z)	229549	000112-11-8	59
2 i-Propyl 9-octadecenoate (Z)	229550	000112-11-8	59
3 n-Propyl 9-octadecenoate	229543	1000336-71-6	49

Unknown Spectrum based on Apex

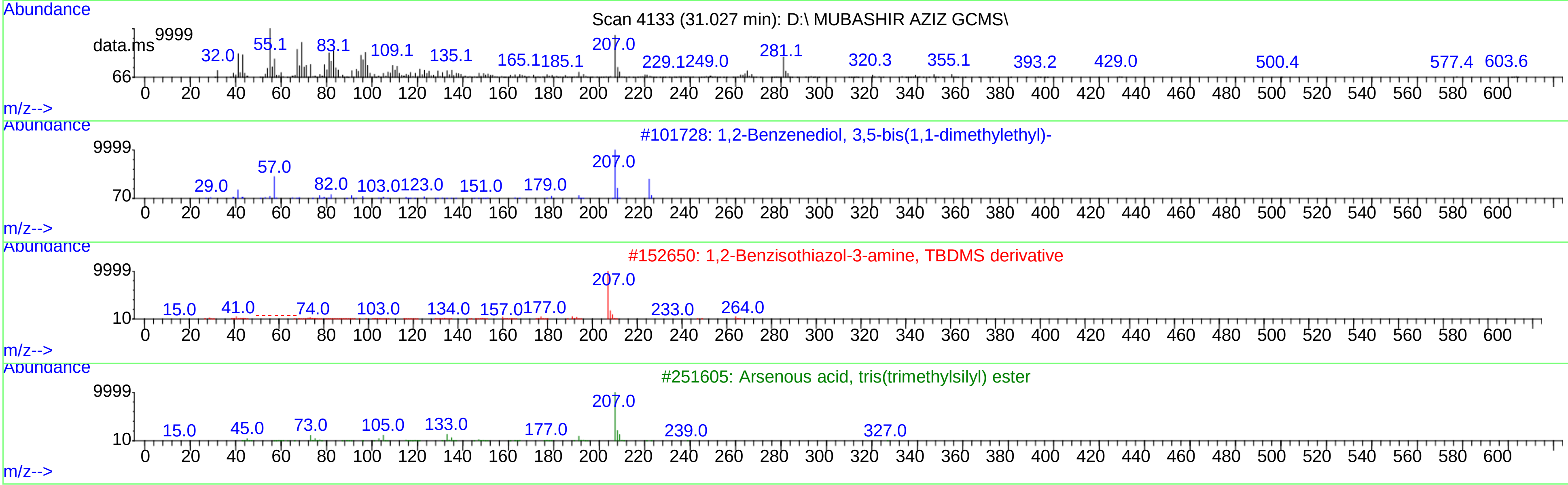

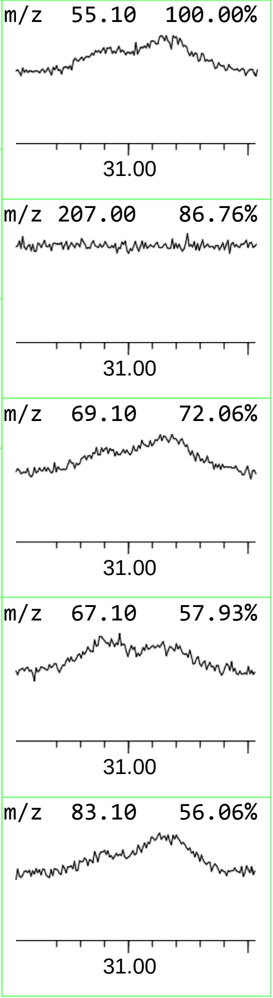

Data File: D:\ MUBASHIR AZIZ GCMS\ DATA
Sample : Pb Cr

Peak Number: 22 at 31.029 min Area: 1436601 Area % 0.66

The 3 best hits from each library. Ref\# CAS\# Qual

C:\MassHunter\LIBRARY\NIST20.L

		Ref\#	CAS\#	Qual
1	1,2-Benzenediol, 3,5-bis(1,1-dim...	101728	001020-31-1	42
2	1,2-Benzisothiazol-3-amine, TBDM...	152650	1000332-57-2	42
3	Arsenous acid, tris(trimethylsil...	251605	055429-29-3	30

Unknown Spectrum based on Apex

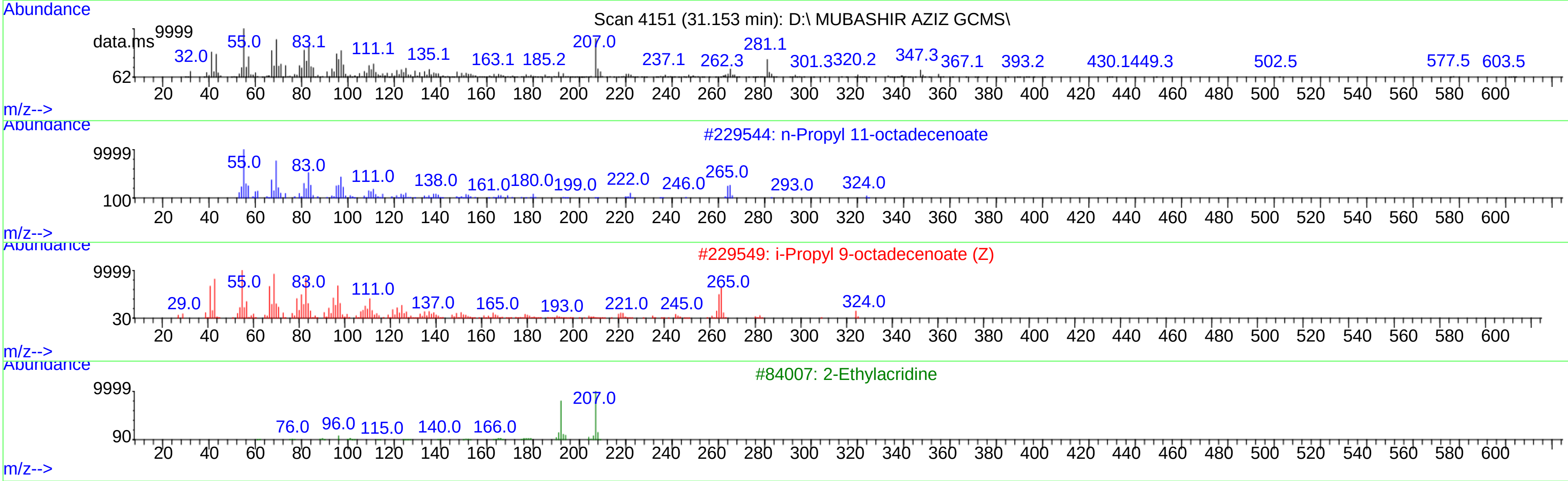

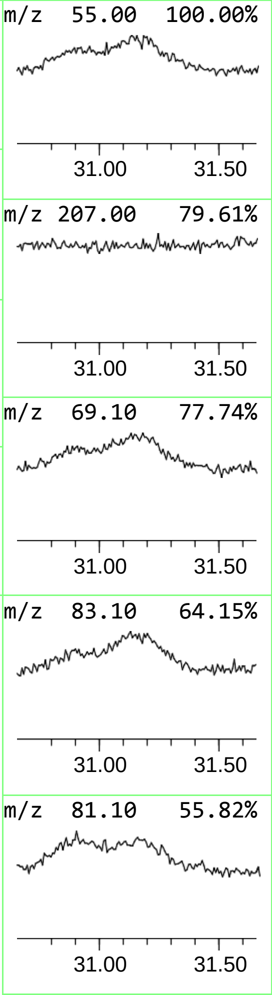

Data File: D:\ MUBASHIR AZIZ GCMS\ DATA
Sample : Pb Cr

Peak Number: 23 at 31.156 min Area: 8483170 Area % 3.92

The 3 best hits from each library. Ref\# CAS\# Qual

C:\MassHunter\LIBRARY\NIST20.L

		Ref\#	CAS\#	Qual
1	n-Propyl 11-octadecenoate	229544	1000336-71-7	43
2	i-Propyl 9-octadecenoate (Z)	229549	000112-11-8	43
3	2-Ethylacridine	84007	055751-83-2	25

Unknown Spectrum based on Apex

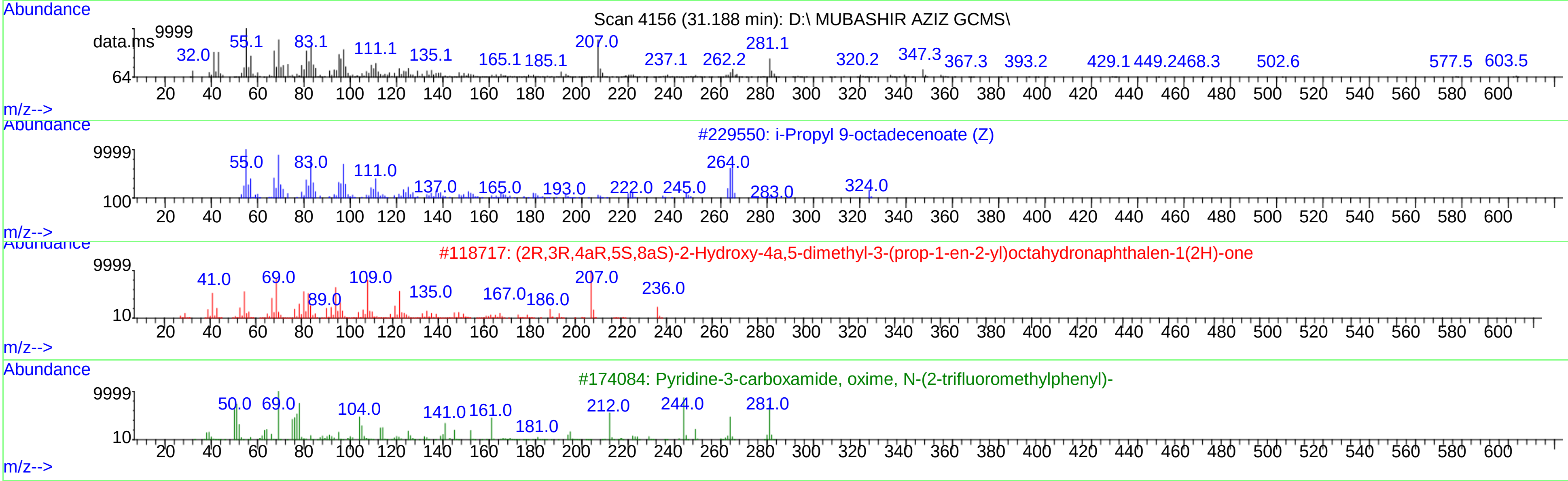

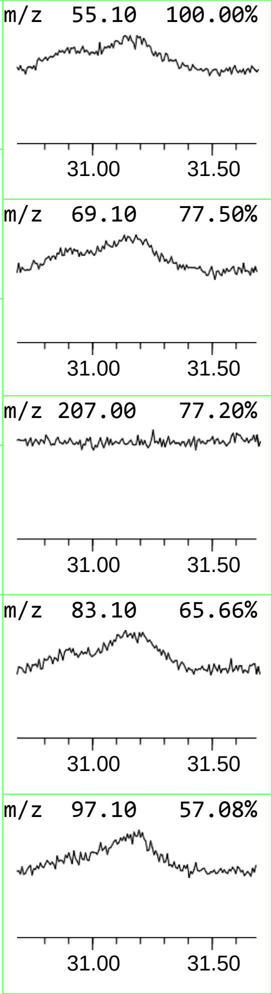

Data File: D:\ MUBASHIR AZIZ GCMS\ DATA
Sample : Pb Cr

Peak Number: 24 at 31.185 min Area: 9399872 Area % 4.35

The 3 best hits from each library.

C:\MassHunter\LIBRARY\NIST20.L

	Name	Ref\#	CAS\#	Qual
1	i-Propyl 9-octadecenoate (Z)	229550	000112-11-8	84
2	(2R,3R,4aR,5S,8aS)-2-Hydroxy-4a,...	118717	066884-74-0	58
3	Pyridine-3-carboxamide, oxime, N...	174084	288246-53-7	42
