# Supplementary material for: Exploration of the anti-inflammatory potential of Polygonum bistorta L.: protection against LPS-induced acute lung injury in rats via NF-ĸβ pathway inhibition
Source: Front Pharmacol. 2025 Feb 5;15:1500085. doi: 10.3389/fphar.2024.1500085 (PMC11851016; doi:10.3389/fphar.2024.1500085)
Supplement: Supplementary file 2 [file DataSheet1.pdf]

Supplementary Material

Research Article

Exploration of the Anti-oxidative and Anti-inflammatory Potential of the *Polygonum bistorta* Linn.: Protection Against LPS-Induced Acute Lung Injury in Rats via NF-κβ Pathway Inhibition

Sajida Parveen[1*], Kashif ur Rehman Khan[2], Shahid Muhammad Iqbal[1], Hanan Y. Aati[3], Areej M. Al-taweel[3], Liaqat Hussain[4], Musaddique Hussain[1,5]

Correspondence: sajida.sajal@gmail.com; musaddique.hussain@iub.edu.pk

Department of Pharmacology, Faculty of Pharmacy, The Islamia University of Bahawalpur, Pakistan.

Table S1: List of compounds with their binding affinities and interactions of docked compounds isolated from *Pb.Cr* against AChE.

Sr. #	Compound/Ligand	Pub Chem CID	Binding Affinity	Amino Acids	Types of Interaction
1.	Phthalic Acid	1017	-6.1	V323/V400/U327/H440/P326/Y441/N324	Alkyl/Pi-Sigma/Unfavorable Doner-Doner /Unfavorable Doner-Doner/H-Bond/H-Bond/H-Bond
2.	1,2-benzenediol-3,5-bis(1,1-dimethylethyl)-	66099	-5.8	F330/F331/T84	Pi-Sigma/Pi-Alkyl/Pi-Alkyl
3.	1,2-Benzisothiazol-3-amine	89966	-7.8	H390/N525/O232/O529/H406	H-Bond/H-Bond/Pi-Alkyl/Pi-Alkyl/Alkyl

4.	10E,12Z-Octadecadienoic acid	5281125	-8.0	N121/T85/F290/F330/F311/H440/T84	H-Bond/H-Bond/ Pi-Alkyl/Pi-Alkyl/ Pi-Alkyl/Pi-Alkyl/ Pi-Alkyl
5.	Oleic acid	445639	-7.4	R121/N85/T84/H440/F290/F330/F331	H-Bond/H-Bond/ Pi-Alkyl/Pi-Alkyl /Pi-Alkyl/Pi-Alkyl /Pi-Alkyl
6.	CIS-Vaccenic acid	5282761	-6.9	T84/F330/F331/H440/Y441/Y117/Y118 /Y119/R121/S122/S200/F290/H440	Pi-Alkyl/Pi-Alkyl/ Pi-Alkyl/Pi-Alkyl /Vander wall's/ Vander wall's/ Vander wall's/ Vander wall's/ Vander wall's/ Vander wall's/ Vander wall's/ Vander wall's/ Vander wall's
7.	7-pentadecyle	549063	-7.5	C231/S235/O229/N230/O232/T233/A234/L305/V236/S304/U306	H-Bond/H-Bond/ H-Bond/Vander wall's/H-bond/ Vander wall's/ Vander wall's/ Alkyl/Vander wall's/Vander wall's/Vander wall's
8.	Cyclohexane Carboxylic acid	221666	-8.6	F331/R121/H440/I69	Pi-Pi-T Shaped/H-Bond/C-H Bond/ C-H Bond

Note: A=Alanine, C=Cystine, F=Phenylalanine, H=Histidine, I=Glutamine, L=Leucine, N=Asparagine, O=Proline, P=Aspartic acid, R=Tyrosine, S=Serine, T=Tryptophane, U=Glutamine, V=Valine, Y=Glycine

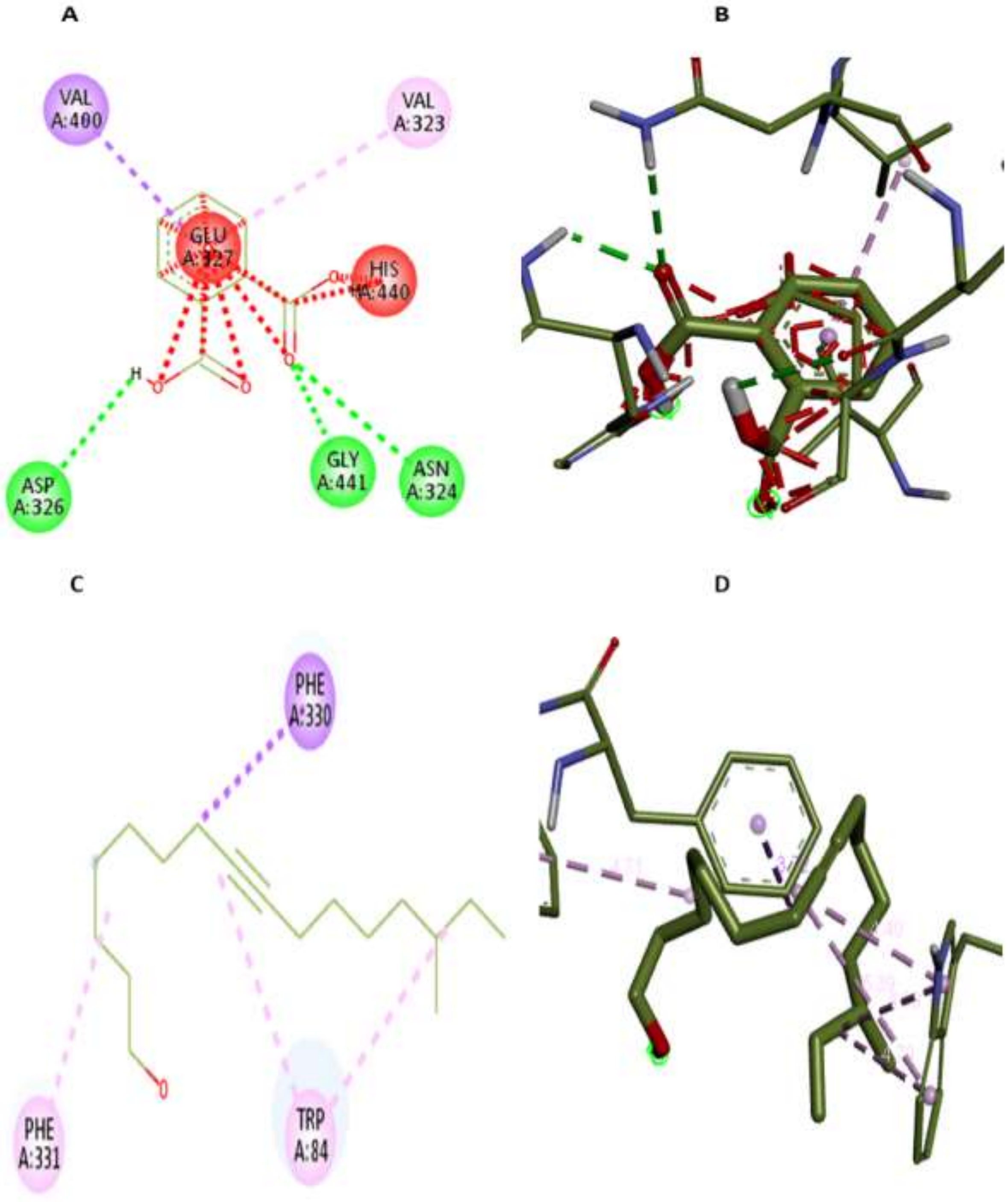

E

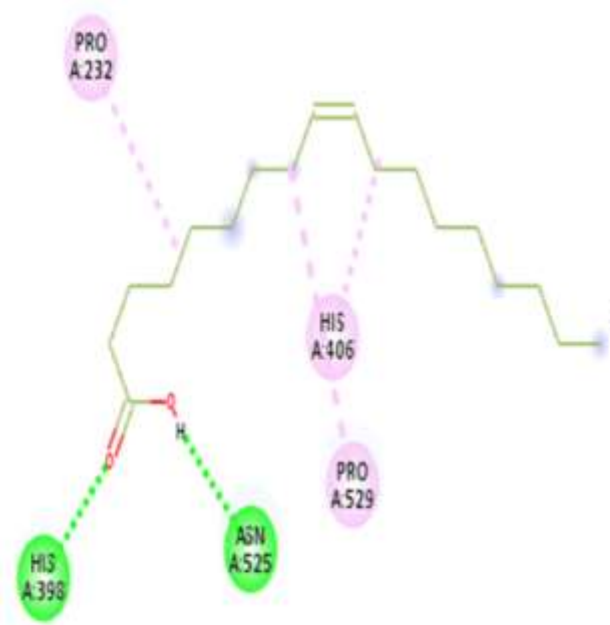
PRO A:232
HIS A:406
PRO A:529
HIS A:398
ASN A:525

F

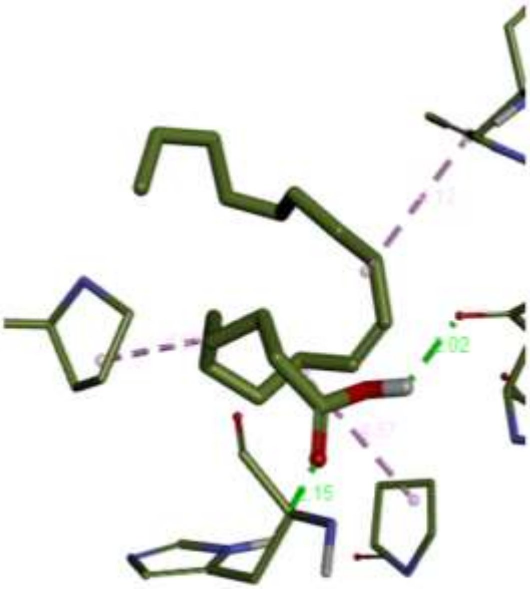

G

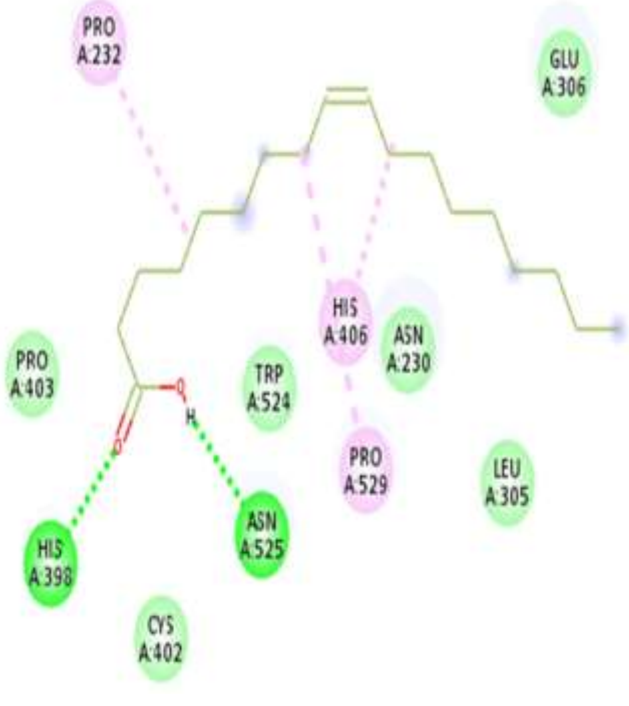
PRO A:232
GLU A:306
HIS A:406
ASN A:230
PRO A:403
TRP A:524
PRO A:529
LEU A:305
HIS A:398
ASN A:525
CYS A:402

H

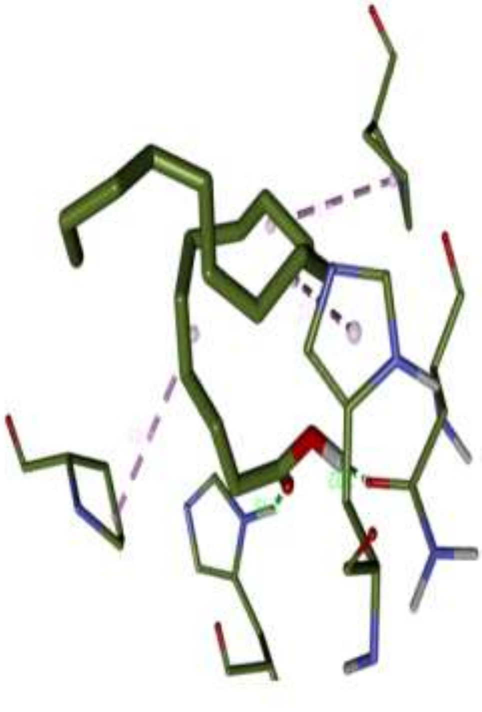

I

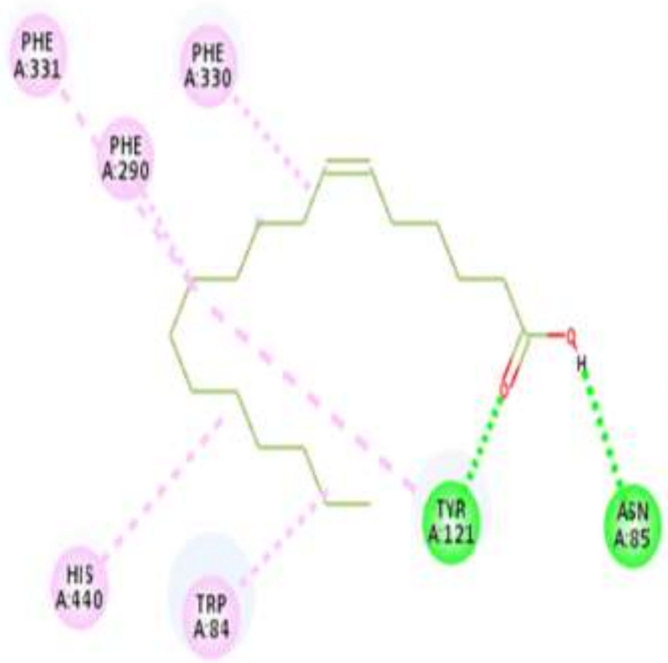
PHE
A:331
PHE
A:330
PHE
A:290
TYR
A:121
ASN
A:85
HIS
A:440
TRP
A:84

J

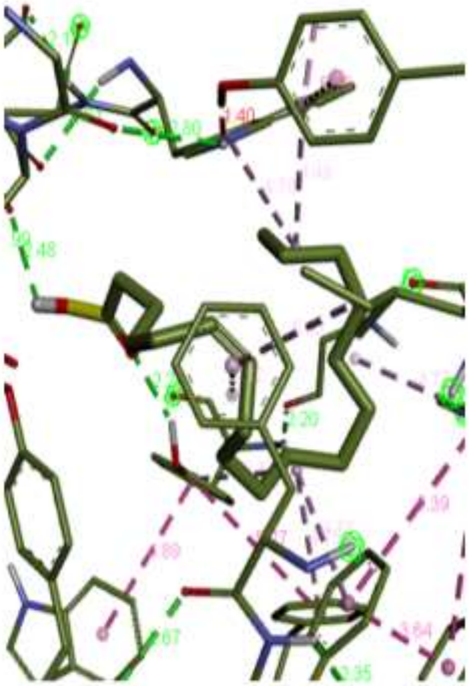

K

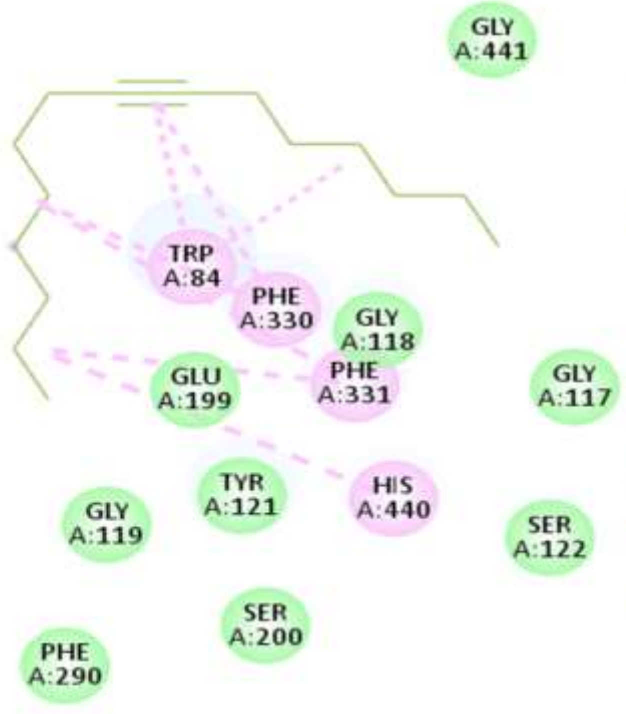
GLY
A:441
TRP
A:84
PHE
A:330
GLY
A:118
GLU
A:199
PHE
A:331
GLY
A:117
TYR
A:121
HIS
A:440
GLY
A:119
SER
A:122
SER
A:200
PHE
A:290

L

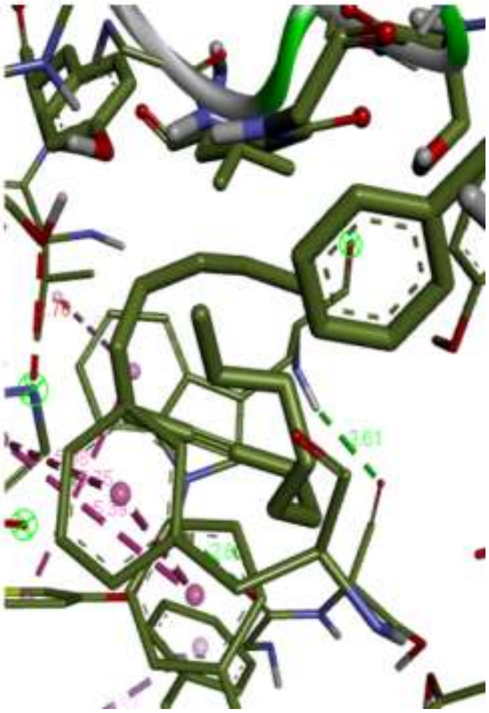

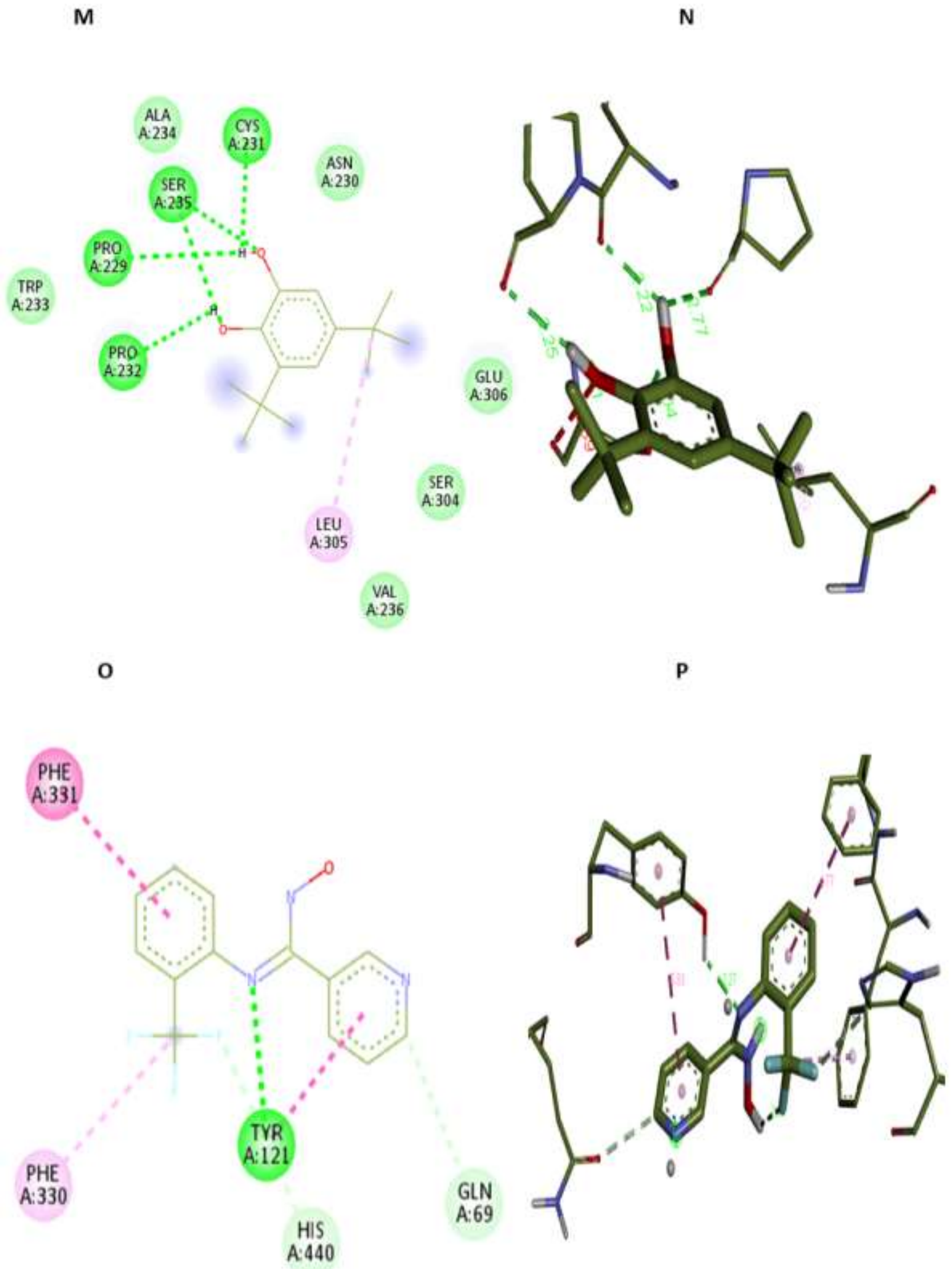
M
N
ALA
A:234
CYS
A:231
ASN
A:230
SER
A:235
PRO
A:229
TRP
A:233
PRO
A:232
GLU
A:306
SER
A:304
LEU
A:305
VAL
A:236
O
P
PHE
A:331
PHE
A:330
TYR
A:121
HIS
A:440
GLN
A:69

Figure: S1. Docking of Phthalic Acid, 1,2-benzenediol, 1,2-Benzisothiazol-3-amine, 10E,12Z-Octadecadienoic acid , Oleic acid, CIS-Vaccenic acid, 7-pentadecyle, Cyclohexane Carboxylic acid interacting with acetyl cholinesterase. (A) Phthalic Acid, 2d (B) 3d (C) 1,2-benzenediol,2d (D) 3d (E) 1,2-Benzisothiazol-3-amine, 2d (F) 3d (G) 10E,12Z-Octadecadienoic acid, 2d (H) 3d (I) Oleic acid, 2d (J) 3d (K) CIS-Vaccenic acid,2d (L) 3d (M) 7-pentadecyle, 2d (N) 3d (O) Cyclohexane Carboxylic acid, 2d (P) 3d compounds.
